# Supplementary material for: Electrochemical ohmic memristors for continual learning
Source: Nat Commun. 2025 Mar 8;16:2348. doi: 10.1038/s41467-025-57543-w (PMC11890563; doi:10.1038/s41467-025-57543-w)
Supplement: Supplementary file 1 — Supplementary Information [file 41467_2025_57543_MOESM1_ESM.pdf]

Supplementary Information for

Electrochemical ohmic memristors for continual learning

Shaochuan Chen[1,2,#,*], Zhen Yang[3,#], Heinrich Hartmann[4], Astrid Besmehn[4], Yuchao Yang[3,5,6,7*], Ilia Valov[8,9*]

[1]Institute of Materials in Electrical Engineering 2 (IWE2), RWTH Aachen University, Aachen, Germany.

[2]International Center for Young Scientists (ICYS), National Institute for Materials Science (NIMS), Tsukuba, Japan.

[3]Beijing Advanced Innovation Center for Integrated Circuits, School of Integrated Circuits, Peking University, Beijing, China.

[4]Central Institute for Engineering, Electronics and Analytics (ZEA-3), Forschungszentrum Jülich, Jülich, Germany.

[5]Guangdong Provincial Key Laboratory of In-Memory Computing Chips, School of Electronic and Computer Engineering, Peking University, Shenzhen 518055, China

[6]Center for Brain Inspired Chips, Institute for Artificial Intelligence, Peking University, Beijing, China.

[7]Center for Brain Inspired Intelligence, Chinese Institute for Brain Research (CIBR), Beijing, China.

[8]Peter Grünberg Institute 7 and JARA-FIT, Forschungszentrum Jülich, Jülich, Germany.

[9]Institute of Electrochemistry and Energy Systems, Bulgarian Academy of Sciences, "Acad. G Bonchev" str. Bl.10, 1113 Sofia, Bulgaria

[#]These authors contributed equally to this work.

*Email: CHEN.Shaochuan@nims.go.jp; yuchaoyang@pku.edu.cn; i.valov@fz-juelich.de

Supplementary Note 1

Charge transport at the electrode/switching layer interface

To analyze the charge transport and electrical characteristics at the metal/oxide interface (Fig. 2, e,f), the overall current density flow in the system is considered as the sum of thermionic-emission current density and the ionic current density

$$j = j_{TE} + j_{ION} \quad \text{(S1)}$$

The Schottky-emission current density is expressed as

$$j_{TE} = j_S \left[\exp\left(\frac{e\Delta\varphi}{kT}\right) - 1\right] \quad \text{(S2)}$$

where $j_S = A^* T^2 \exp\left(-\frac{e\phi_B}{kT}\right)$ is the saturation current density, A^* is the effective Richardson constant, T is the absolute temperature, e is the elementary charge, ϕ_B is the barrier height, k is the Boltzmann constant, $\Delta\varphi$ is the applied voltage.

The ionic-redox current density is expressed using the Butler-Volmer equation

$$j_{ION} = j_0 \left[\exp\left(\frac{\alpha_a z e\Delta\varphi}{kT}\right) - \exp\left(-\frac{\alpha_c z e\Delta\varphi}{kT}\right)\right] \quad \text{(S3)}$$

where $j_0 = zek_0c_0 \exp\left(-\frac{\Delta G_a}{kT}\right)$ is the exchange current density, z is the charge number, k_0 is the rate constant, c_0 is the concentration of ions at equilibrium, ΔG_a is the free energy of activation, α_a is the anodic transfer coefficient, $\alpha_c = 1 - \alpha_a$ is the cathodic transfer coefficient, $\Delta\varphi$ is the electron−transfer overpotential.

Assume the charge number $z = 2$, anodic and cathodic transfer coefficient is identical, i.e., $\alpha_a = \alpha_c = 0.5$, then the overall current density

$$j = j_S \left[\exp\left(\frac{e\Delta\varphi}{kT}\right) - 1\right] + j_0 \left[\exp\left(\frac{e\Delta\varphi}{kT}\right) - \exp\left(-\frac{e\Delta\varphi}{kT}\right)\right]$$

$$= j_S \left[\exp\left(\frac{e\Delta\varphi}{kT}\right)\right] - j_S + j_0 \left[2\sinh\left(\frac{e\Delta\varphi}{kT}\right)\right] \quad \text{(S4)}$$

Now we consider if the Schottky barrier height $e\phi_B$ in the system is sufficiently high, $\left[\exp\left(-\frac{e\phi_B}{kT}\right)\right] \approx 0$, saturation current $j_S = A^* T^2 \exp\left(-\frac{e\phi_B}{kT}\right) \approx 0$ (in the case that the reverse bias current is not affected by image-force barrier lowering), Equation (S4) is modified as

$$j = j_S \left[\exp\left(\frac{e\Delta\varphi}{kT}\right)\right] + j_0 \left[2\sinh\left(\frac{e\Delta\varphi}{kT}\right)\right] \quad \text{(S5)}$$

(i) When the system is positively biased, $\Delta\varphi > 0$, consider the applied voltage is much higher than the thermal voltage: $\Delta\varphi \gg \frac{kT}{e}$, we have $\frac{e\Delta\varphi}{kT} \gg 1$, thus $\left[2\sinh\left(\frac{e\Delta\varphi}{kT}\right)\right] \approx \exp\left(\frac{e\Delta\varphi}{kT}\right)$, Equation S5 can be modified as

$$j = j_S\left[\exp\left(\frac{e\Delta\varphi}{kT}\right)\right] + j_0\left[\exp\left(\frac{e\Delta\varphi}{kT}\right)\right] \tag{S6}$$

j can be further written as

$$j = A^*T^2\left[\exp\left(\frac{\Delta\varphi - \phi_B}{kT}e\right)\right] + 2ekc\left[\exp\left(\frac{e\Delta\varphi - \Delta G_a}{kT}\right)\right] \tag{S7}$$

We attribute this condition to the region I in Fig. 2e. The energy diagram is presented in Fig. 2a. The current density is the sum of Schottky-emission current density (forward-biased) and ionic current density.

(ii) When the system is under reversed biased, $\Delta\varphi < 0$, consider the absolute value of applied voltage is high and much higher than the thermal energy, then $-\frac{e\Delta\varphi}{kT} \gg 1$, thus $\left[2\sinh\left(\frac{e\Delta\varphi}{kT}\right)\right] \approx -\exp\left(-\frac{e\Delta\varphi}{kT}\right)$, Equation S5 is modified as

$$j = -j_0\exp\left(-\frac{e\Delta\varphi}{kT}\right) \tag{S8}$$

j can be further written as

$$j = -2ekc\exp\left(\frac{-e\Delta\varphi - \Delta G_a}{kT}\right) \tag{S8}$$

We attribute this condition to region II in Fig. 2e. The energy diagram is presented in Fig. 2b. The current density is dominated by ionic current density.

Now we consider if the Schottky barrier height $e\phi_B$ in the redox system is low, compared to cases i) and ii), but cannot be approximated as zero. Saturation currents j_S cannot be neglected, Equation S4 is kept as

$$j = j_S\left[\exp\left(\frac{e\Delta\varphi}{kT}\right) - 1\right] + j_0\left[\exp\left(\frac{e\Delta\varphi}{kT}\right) - \exp\left(-\frac{e\Delta\varphi}{kT}\right)\right] \tag{S9}$$

(iii) When the system is positively biased, $\Delta\varphi > 0$, consider the applied voltage is much higher than the thermal voltage: $\Delta\varphi \gg \frac{kT}{e}$, similar to (i), $\frac{e\Delta\varphi}{kT} \gg 1$, $\left[2\sinh\left(\frac{e\Delta\varphi}{kT}\right)\right] \approx \exp\left(\frac{e\Delta\varphi}{kT}\right)$,

Equation S9 is modified as

$$j = j_S\left[\exp\left(\frac{e\Delta\varphi}{kT}\right) - 1\right] + j_0\left[\exp\left(\frac{e\Delta\varphi}{kT}\right)\right] \tag{S10}$$

j can be further written as

$$j = A^*T^2\left[\exp\left(\frac{\Delta\varphi - \phi_B}{kT}e\right) - \exp\left(-\frac{e\phi_B}{kT}\right)\right] + 2ekc\left[\exp\left(\frac{e\Delta\varphi - \Delta G_a}{kT}\right)\right] \tag{S11}$$

We attribute this condition to region III in Fig. 2f. The energy diagram is presented in Fig. 2c. The current density is the sum of Schottky-emission current density (forward-biased) and ionic current density. In addition, owing to the low energy barrier, the overall current density in this condition is higher than that in condition (i).

(iv) When the system is under reversed biased, $\Delta\varphi < 0$, consider the absolute value of applied voltage is high and much higher than the thermal energy, similar to (ii), we have $\left[\exp\left(\frac{e\Delta\varphi}{kT}\right)\right] \approx 0$ and $-\frac{e\Delta\varphi}{kT} \gg 1$, $\left[2\sinh\left(\frac{e\Delta\varphi}{kT}\right)\right] \approx -\exp\left(-\frac{e\Delta\varphi}{kT}\right)$, Equation S9 is modified as

$$j = j_S\left[\exp\left(\frac{e\Delta\varphi}{kT}\right) - 1\right] - j_0\left[-\exp\left(-\frac{e\Delta\varphi}{kT}\right)\right] \tag{S12}$$

j can be further written as

$$j = A^*T^2\left[\exp\left(\frac{\Delta\varphi - \phi_B}{kT}e\right) - \exp\left(-\frac{e\phi_B}{kT}\right)\right] - 2ekc\left[\exp\left(\frac{-e\Delta\varphi - \Delta G_a}{kT}\right)\right] \tag{S13}$$

We attribute this condition to region IV in Fig. 2f. The energy diagram is presented in Fig. 2d. The current density is the sum of Schottky-emission current density (reversely biased) and ionic current density. Owing to the low energy barrier, the saturation currents are high in this case, the overall current density are higher than that in condition (ii).

Supplementary Note 2

The effect on neural network performances under different line resistance and cell ratio.

During the binary programming method, the line resistances need to be considered in testing the neural network performance. Considering the square line resistance as R_{line}, the $ratio$ is set as width/length, for one device in the (i,j) position of the memristor array, the overall line resistance R_{all_line} will be

$$R_{all\_line} = j \times R_{line} \times ratio + 2i \times R_{line} \times \frac{1}{ratio}$$

Where $2i$ is induced by the differential cell design. After the pulse programming, the device conductance is G, when considering the line resistance effect, the actual inference weights will be

$$G_{actual} = \frac{1}{\frac{1}{G} + R_{all\_line}} = G \frac{1}{1 + G \times R_{all\_line}}$$

The final results are presented in Supplementary Fig. 36, which provides the design guidance for picking the proper line resistance and cell ratio. For example, in the TSMC 180 nm process node, the line resistance of M1-M5 is 0.08 Ω/square, cell ratio is supposed to be 2.0 to reduce the effect as much as possible.

The effect on neural network performances under different fail ratios.

The electrical properties of ohmic memristors are superior to that of conventional VCMs, especially in the endurance aspect. The continual learning application requires more frequent updates of device conductance, so the endurance property is more critical. To demonstrate the effect of device failure on the final accuracies, we assume that the devices are stuck at LRS (Supplementary Fig. 8), and the possibility of both two devices failing simultaneously is so small to be ignored. The cell weight will be fixed at ±1 or 0 when one of the devices is stuck at LRS, the possibility for weight values of +1, -1 and 0 are 25%, 25% and 50%, respectively. The number of failed devices will accumulate and increase during the continual training, and the failed device position will be kept constantly.

Supplementary Table 1: Intrinsic energy barrier height at metal/Ta_2O_5 interface

Metal	Work function (eV)	Metal-Ta_2O_5 barrier height $e\phi_B$ (eV)
Pt	5.65	2.45 eV
Cu	4.65	1.45 eV
Hf	3.90	0.70 eV
Ta	4.25	1.05 eV
Zr	4.05	0.85 eV

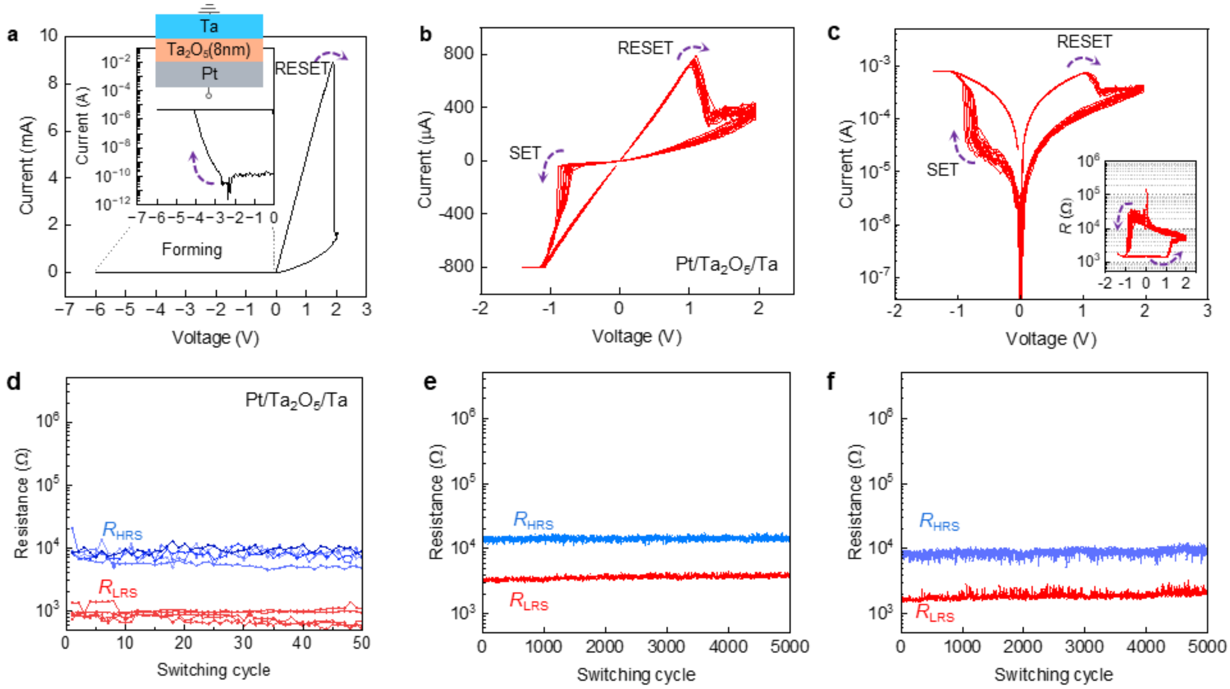

Supplementary Fig. 1. Electrical properties of a conventional VCM device. Exemplary forming (**a**) and resistive switching characteristics (**b**,**c**) of a conventional VCM: Pt/Ta_2O_5/Ta/Pt device. **d**, Endurance test of 5 devices (50 cycles each) showing the high resistive state (HRS) resistance (R_{HRS}) and low resistive state (LRS) resistance (R_{LRS}) values as a function of switching cycles. The R_{HRS}/R_{LRS} is ~10 and shows no distinct degradation after five thousand switching cycles in different devices (**e**,**f**). The resistances at HRS and LRS were read by −200 mV/20 ms voltage pulses. SET pulse: -1 V/20 ms, RESET pulse 1.8 V/40 ms. The voltage amplitudes and stress time are selected to achieve optimal device performance, i.e., high HRS and LRS resistance ratio with high cycle-to-cycle uniformity and high endurance.

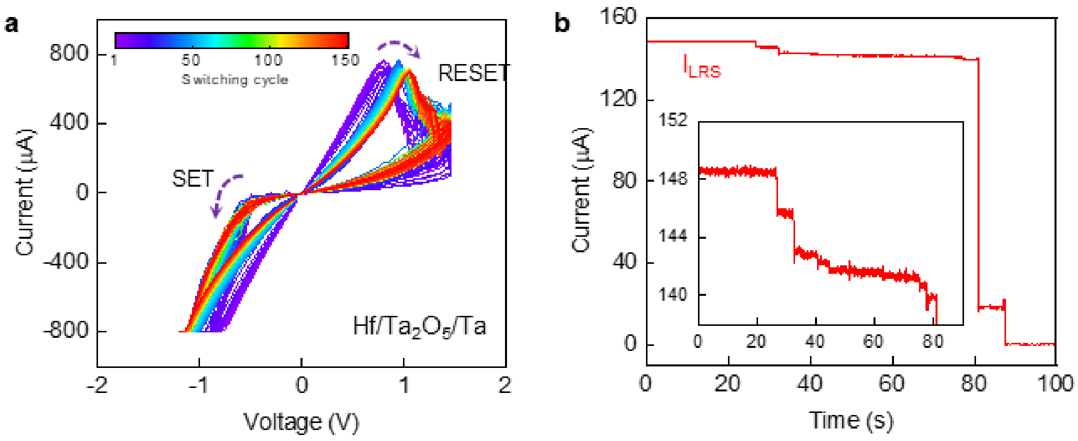

Supplementary Fig. 2. Electrical stability of ohmic memristive device without capping layers. **a**, One hundred and fifty consecutive *I-V* curves from a Hf/Ta_2O_5/Ta device. A clear degradation in cycle-to-cycle uniformity was observed with increasing switching cycles. **b**, Constant voltage stress measurement shows the instability of low resistive state current (I_{LRS}). The I_{LRS} was recorded by applying a constant −200 mV voltage after the SET process. With increasing resistive switching cycles a, or longer read voltage stress time b, the current at LRS is decreasing, indicating the increasing series resistance resulting from the metal electrodes passivation. These reliability problems were also found in all other ohmic memristive devices without or using thin capping layers (APL Mater. 6, 046106, 2018; Adv. Electron. Mater. 5, 1800933, 2019).

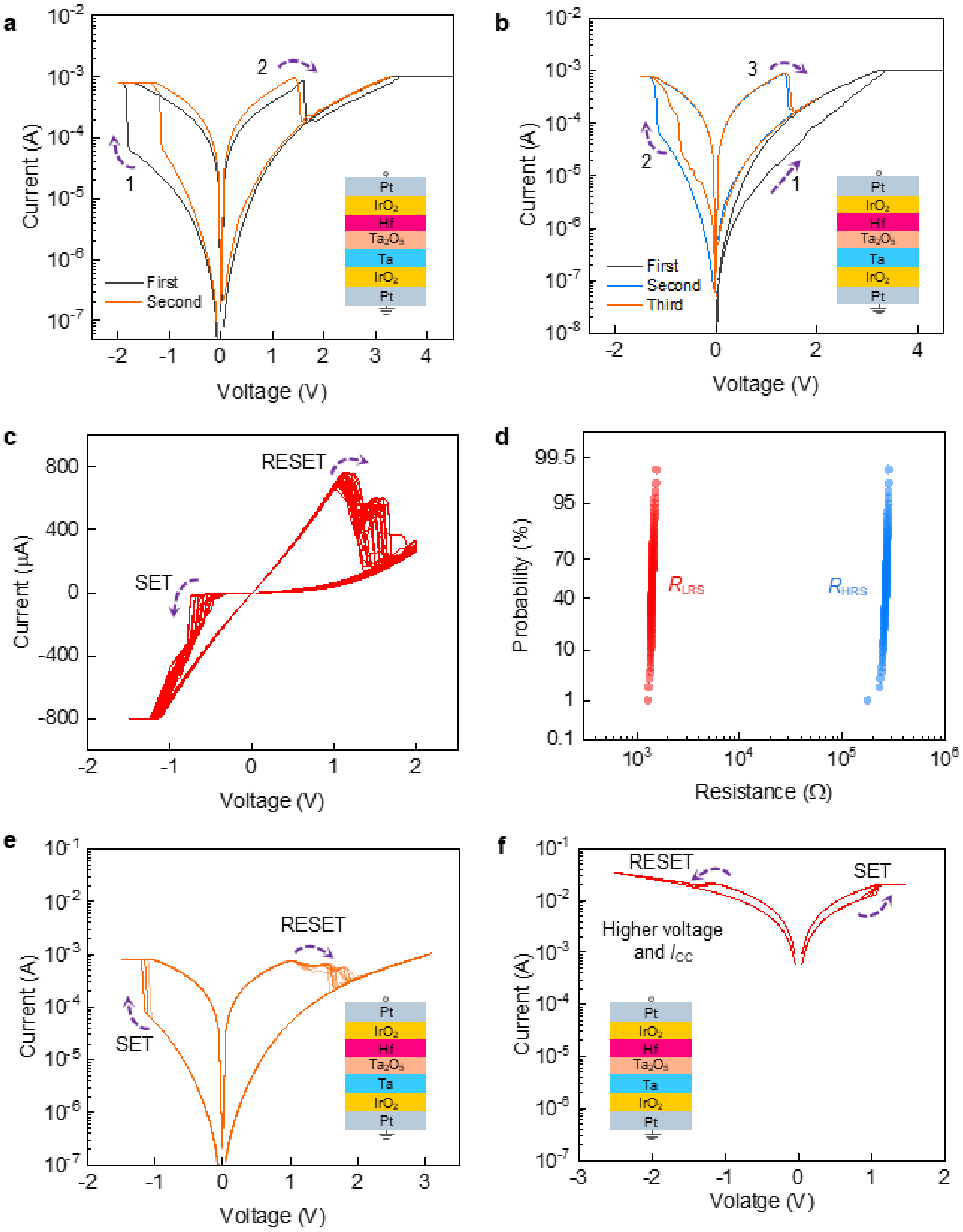

Supplementary Fig. 3. Resistive switching in ohmic memristive device with IrO2/Pt capping layers. a, Forming process and the subsequent *I-V* curves. The device was formed (SET) into LRS by applying negative voltage to top electrode (Hf). **b**, Device operation started with applying positive voltage to Hf electrode (First sweep). The current decreased in the backward sweep, showing no reliable forming was achieved. A successful forming was reached in the second negative voltage sweep. The results show that forming of the devices was successful only for the negative polarization (towards top Hf electrode). In this way the cells were SET into LRS and the RESET process occurs at positive voltages. **c**, Exemplary *I-V* curves of the memristive devices

after forming process. The *I-V* curves are comparable to those in conventional: Pt/Ta_2O_5/Ta/Pt VCM device (Supplementary Fig. 1b,c). **d**, Cumulative probability of resistances at high resistive state and low resistance state (read at 0.2 V) from one hundred *I-V* curves (shown in Fig. 1d). The use of capping layers prevents the passivation of the ohmic electrodes and leads to improved cycling uniformity and electrical stability. **e**, Typical c8w resistive switching (shown in semilogarithmic scale) observed in ohmic memristive device (see also Fig. 1d). **f**, 8w switching polarity observed at high voltage and current ranges. The transition from c8w to 8w switching mode was reached if highly voltage was applied to already formed system. The transition can occur after the first RESET, but can be reached at any cycle of c8w switching after applying high sweep voltages. The c8w to 8w transition is irreversible, and it means device failure. Note that using high voltage or high currents (>10 mA) should be avoided in practical applications, since high voltage/current operation can cause increasing defects in the metal oxide. Here, we show the 8w switching to study the possible failure mechanism.

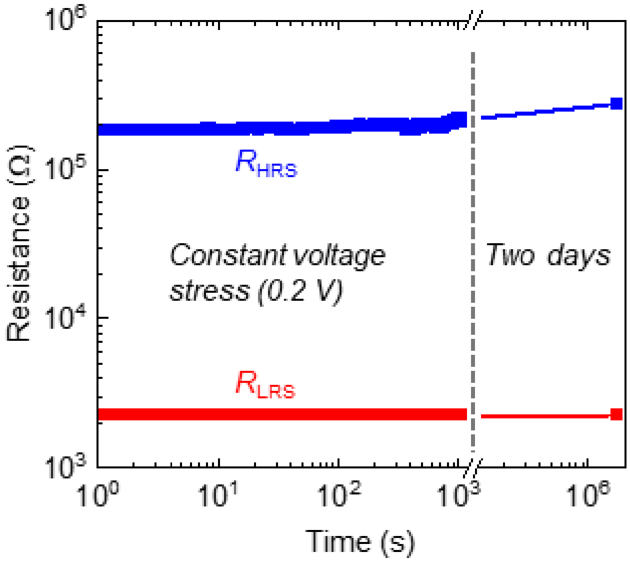

Supplementary Fig. 4. Retention characterization of the ohmic memristive device for over two days.

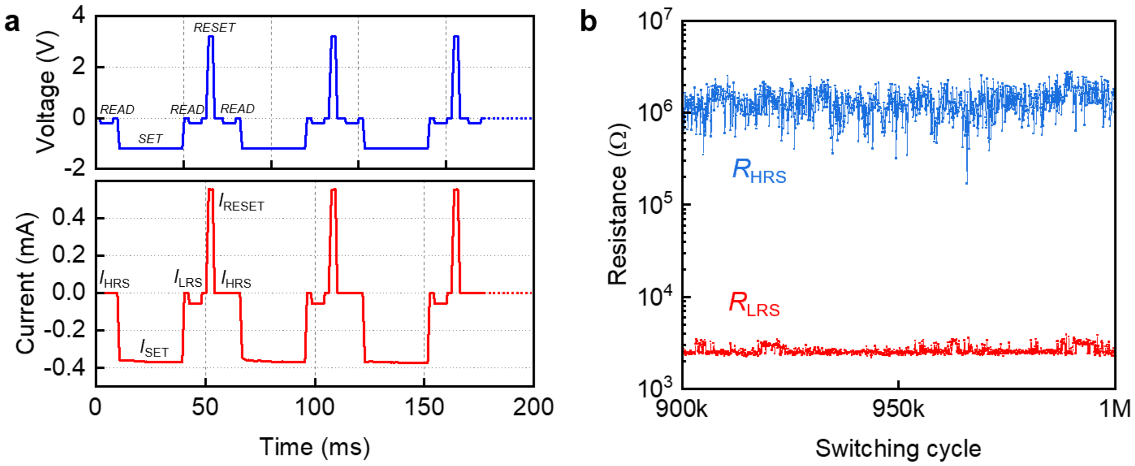

Supplementary Fig. 5. Endurance test. a, The upper panel shows the applied pulse waveform. Each switching cycle contains sequenced READ (HRS)→SET→READ (LRS)→RESET→READ (HRS) operation. The lower panel shows the recorded current signals under the applied pulse voltage stress (V_{READ}: –0.2 V/6 ms, V_{SET}: –1.2 V/30 ms, V_{RESET}: 3.2 V/2 ms). **b**, A zoomed-in view of the endurance plot (Fig. 1e) from switching cycle 9×10^5 to 10^6.

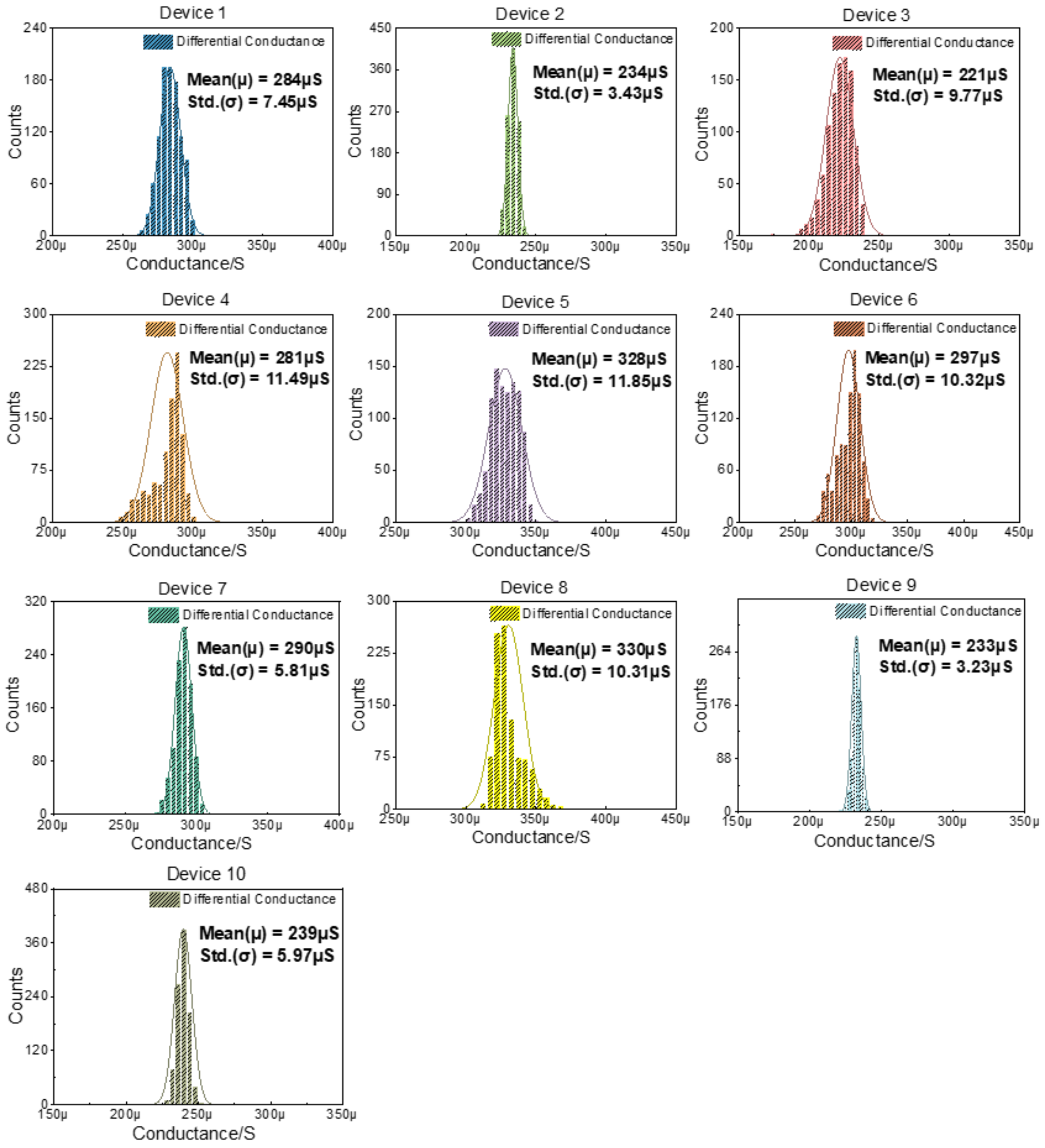

Supplementary Fig. 6. The differential conductance distribution for different devices with 1000 endurance cycles.

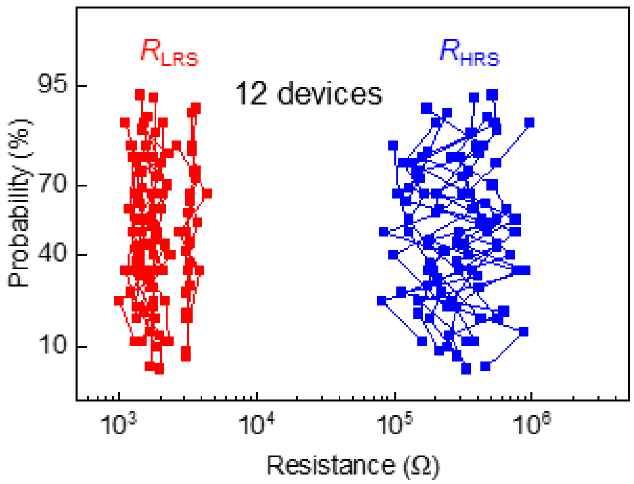

Supplementary Fig. 7. Additional data showing low device-to-device variability of ohmic memristive devices.

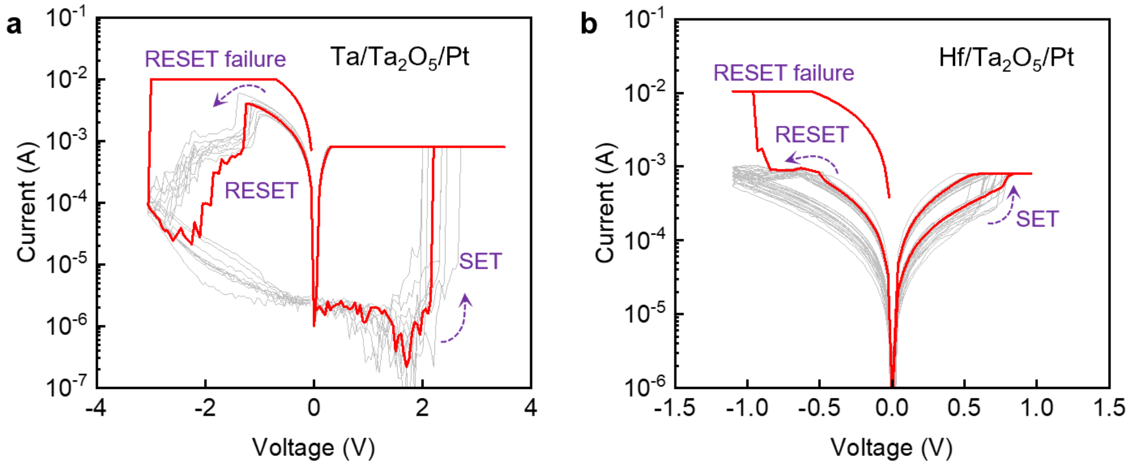

Supplementary Fig. 8. Comparison of electrical characteristics. a, *I-V* characteristics of a $Ta/Ta_2O_5/Pt$ and **b**, $Hf/Ta_2O_5/Pt$ valence change memory. The red curve, which is the 17th IV sweep (a) and 21st IV sweep (b), respectively, shows a typical device failure at the RESET voltage sweep. The sudden increase of the current implies the massive generation of oxygen defects at the Ta_2O_5 layer, making the device stuck at LRS. The high voltage drop across the Schottky-like interface can lead to RESET failure due to the excessive generation of oxygen vacancies. This is also ascribed in part by the low (negative) oxygen vacancy formation energy in Ta_2O_5 layer. For the ohmic memristive devices, no reset failure was observed during the $I-V$ sweeps (under the same operation parameters) and pulse measurements (Fig. 1d,e), indicating an improvement in device reliability in the ohmic memristive system.

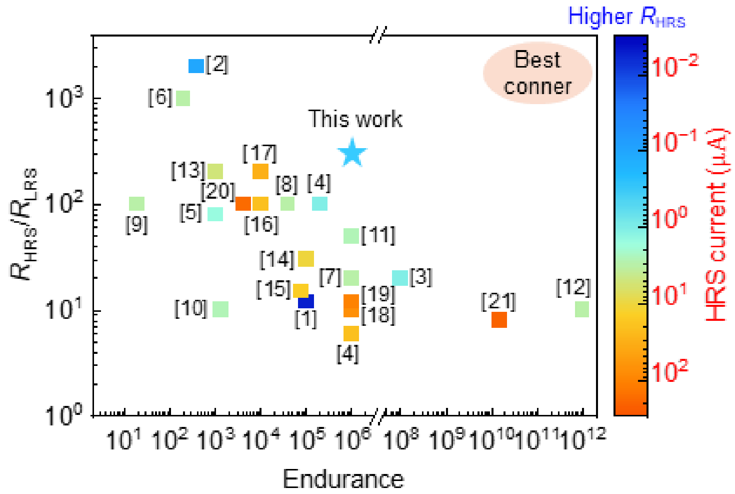

Supplementary Fig. 9. Comparison of endurance, HRS and LRS resistance ratio, overall device current levels with reported VCM devices.

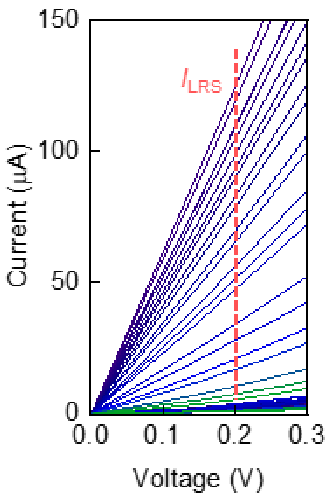

Supplementary Fig. 10. DC IV curves showing multi-bit switching in ohmic memristive devices.

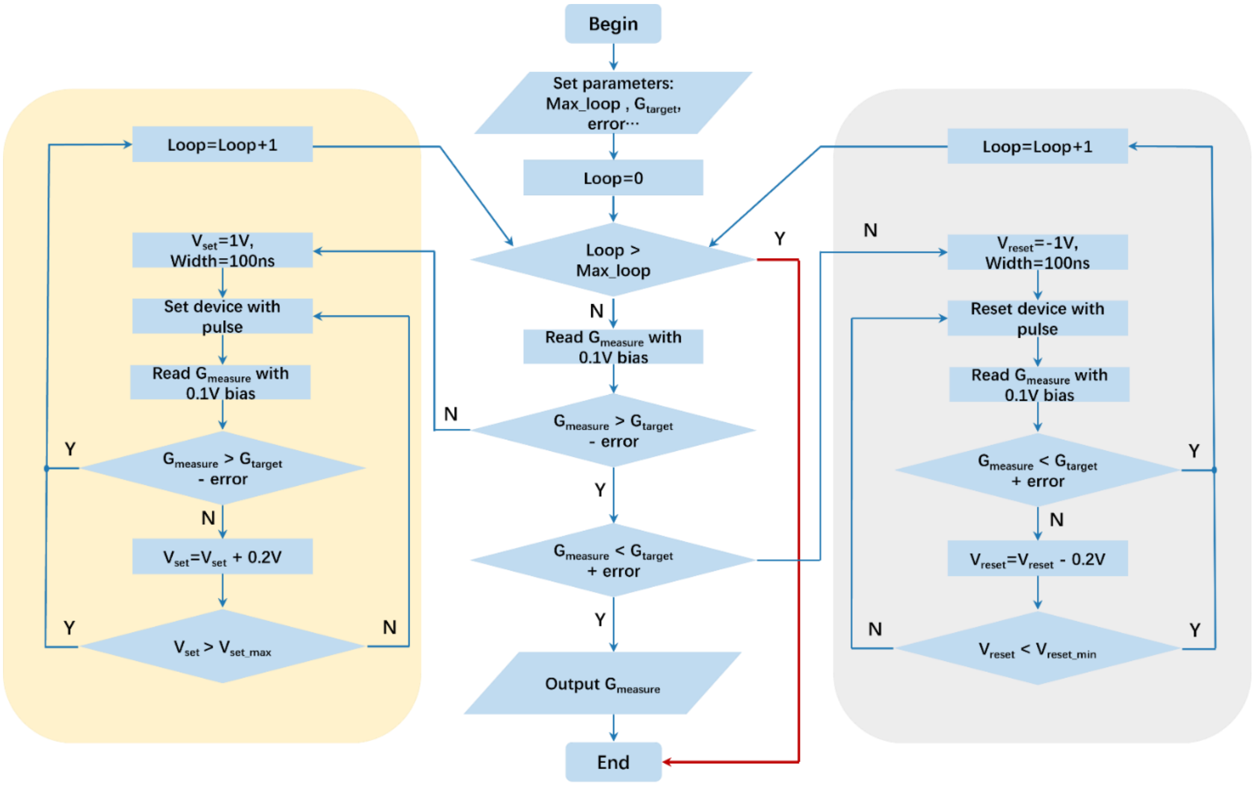

Supplementary Fig. 11. Flowchart of the write-verify programming algorithm. It mainly includes two modules as SET and RESET, both work in negative feedback principle.

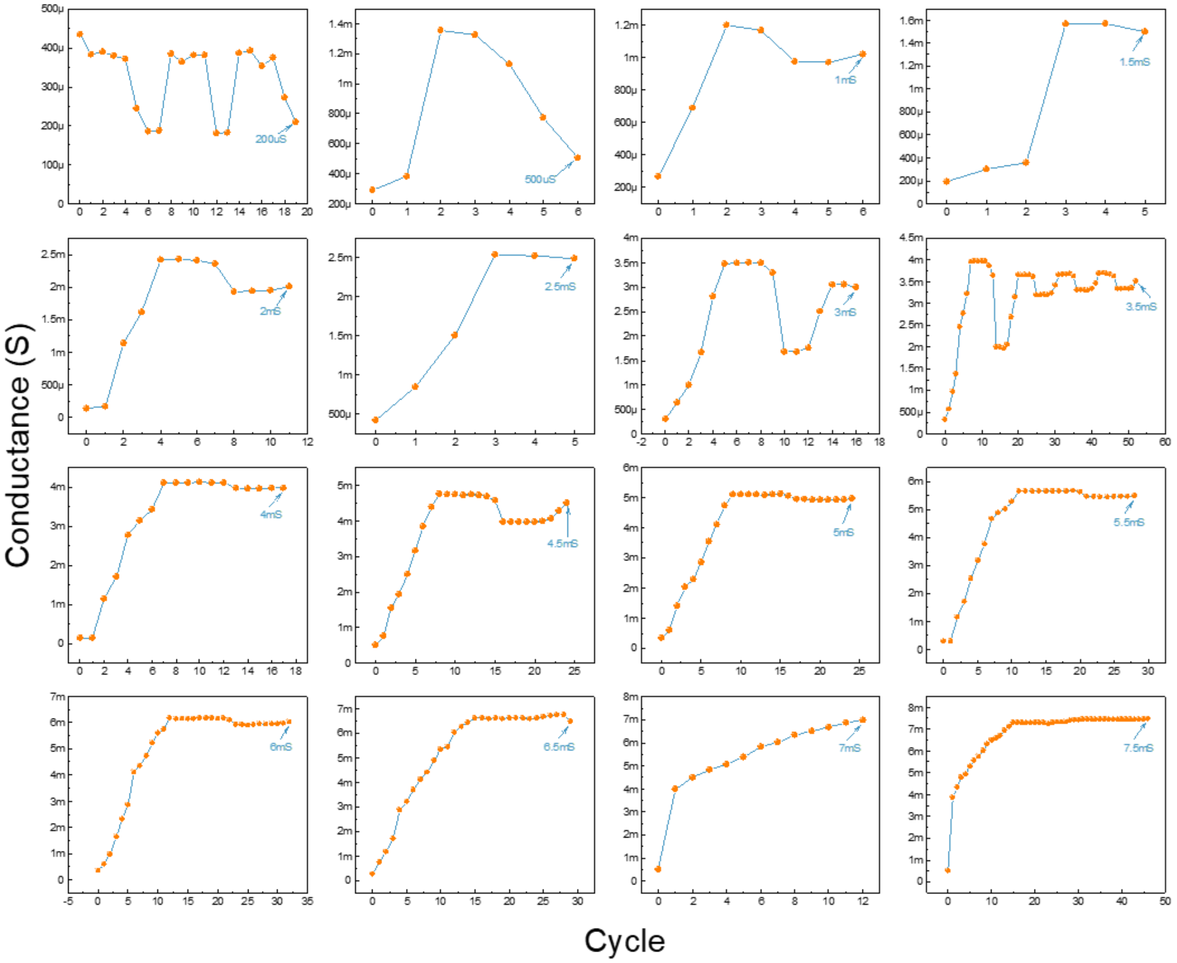

Supplementary Fig. 12. Examples of the device conductance variation under the write-verify scheme.

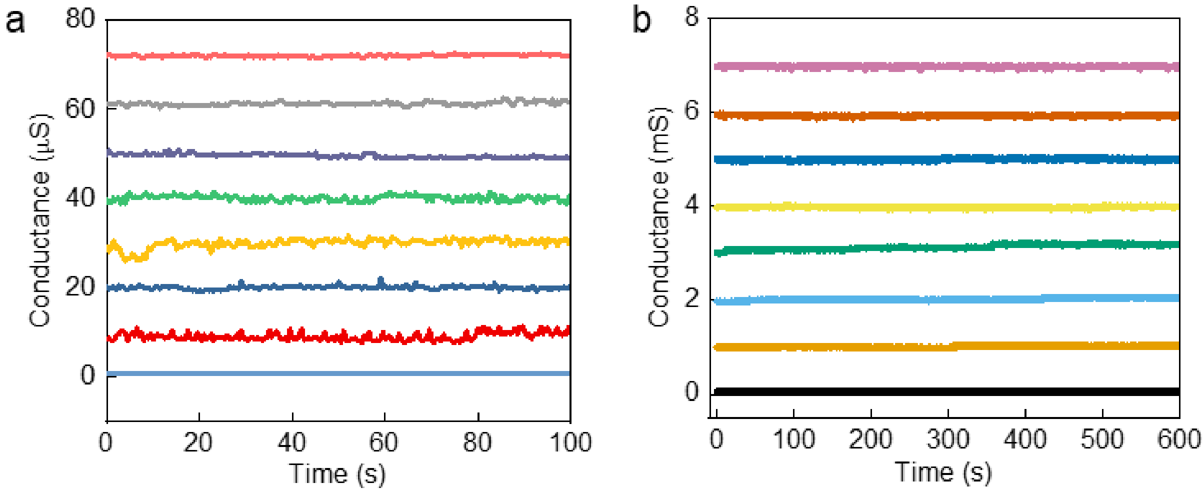

Supplementary Fig. 13. Stability of analogue switching. Retention of multiple LRS conductance levels at µS range (a) and mS range (b) with 0.1 V read bias, all conductance states show no distinguishable degradation for more than 100 s.

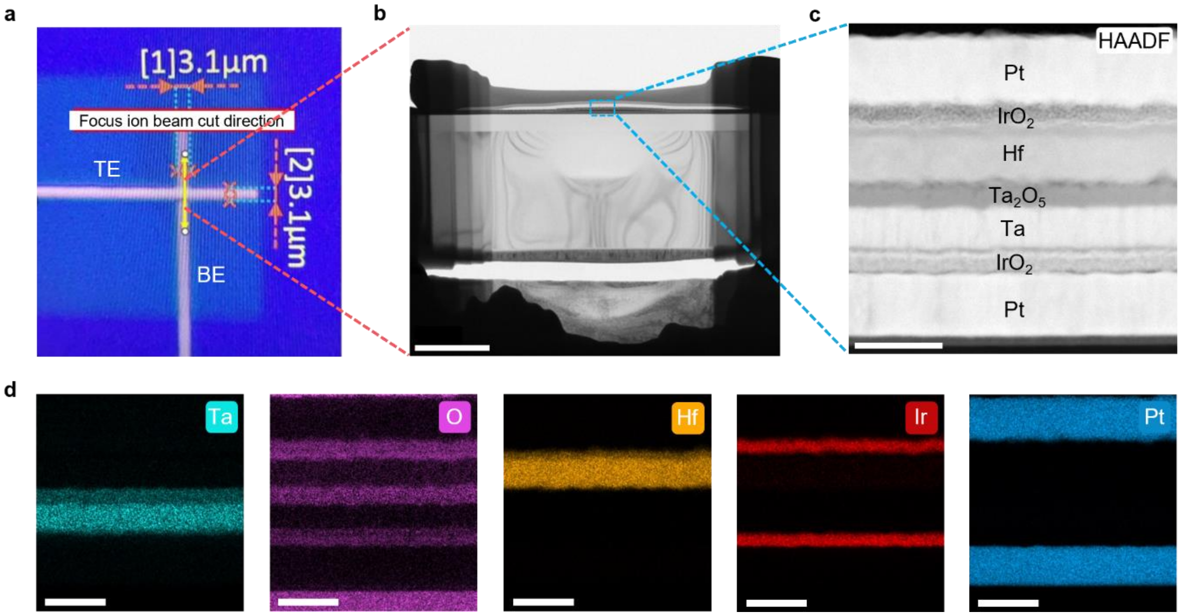

Supplementary Fig. 14. Cross-sectional TEM and energy-dispersive X-ray spectroscopy characterization. a, Schematic of the crossbar intersection and the direction for focus ion beam treatment. **b**, TEM lamella prepared by focus ion beam. Scale bar, 2 μm. **c**, High-angle annular dark-field TEM image showing the stack information. Scale bar, 50 nm. **d**, EDS spectra of each layer. The element species are indicated in the upper right of each panel. Scale bar, 50 nm.

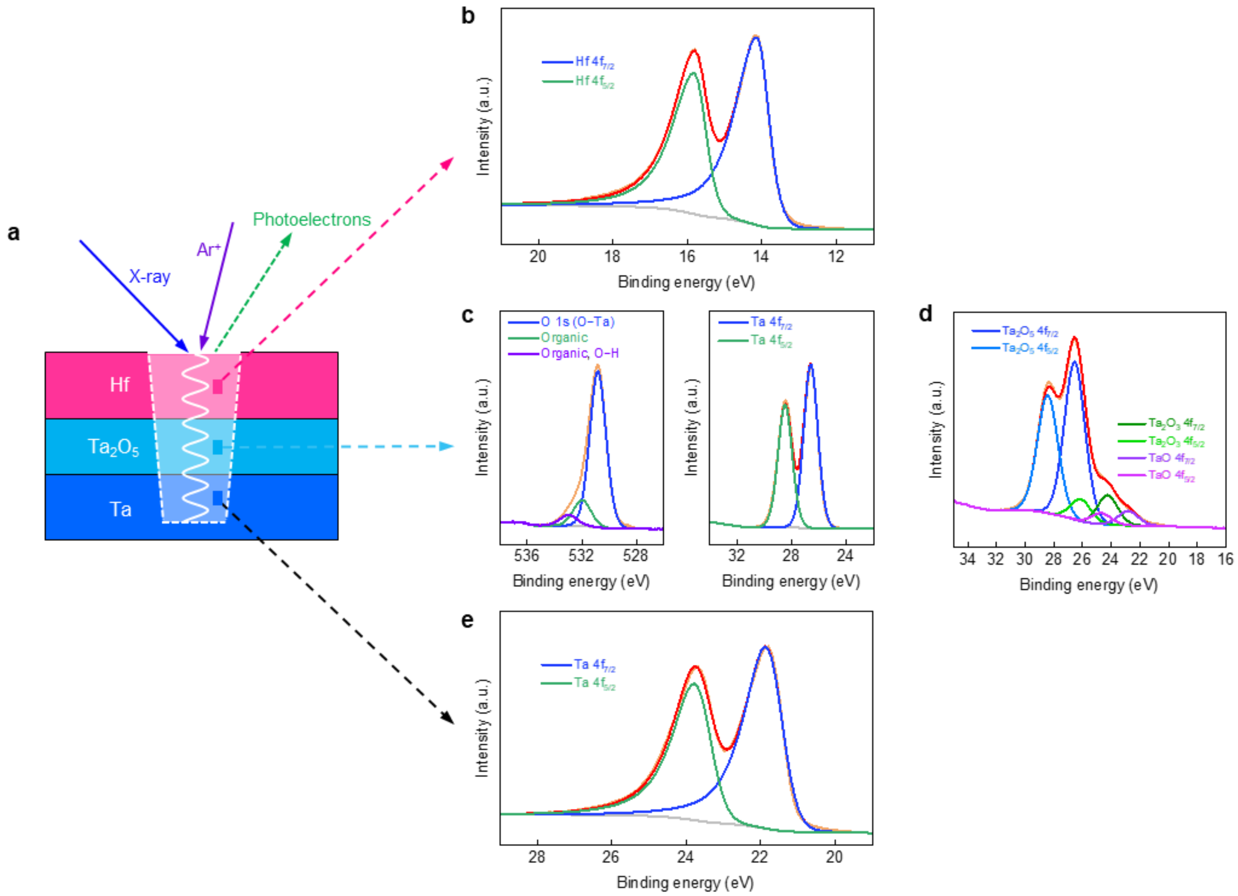

Supplementary Fig. 15. X-ray photoelectron spectroscopy (XPS) depth profiling. a, Schematic illustration of XPS depth profiling performed on the Hf/Ta_2O_5/Ta device. By using monatomic Ar^+ as a sputtering source it allows to obtain vertical chemical profiles of the memristive device. The results reveal the presence of metallic Hf component (**b**), stoichiometric Ta_2O_5 (**c**) and different compositions (d), and metallic Ta component (**e**). We also performed XPS depth profiling on Ta/Ta_2O_5/Ta, Zr/Ta_2O_5/Ta samples, all results reveal the metallic phase of the top electrode and bottom electrode.

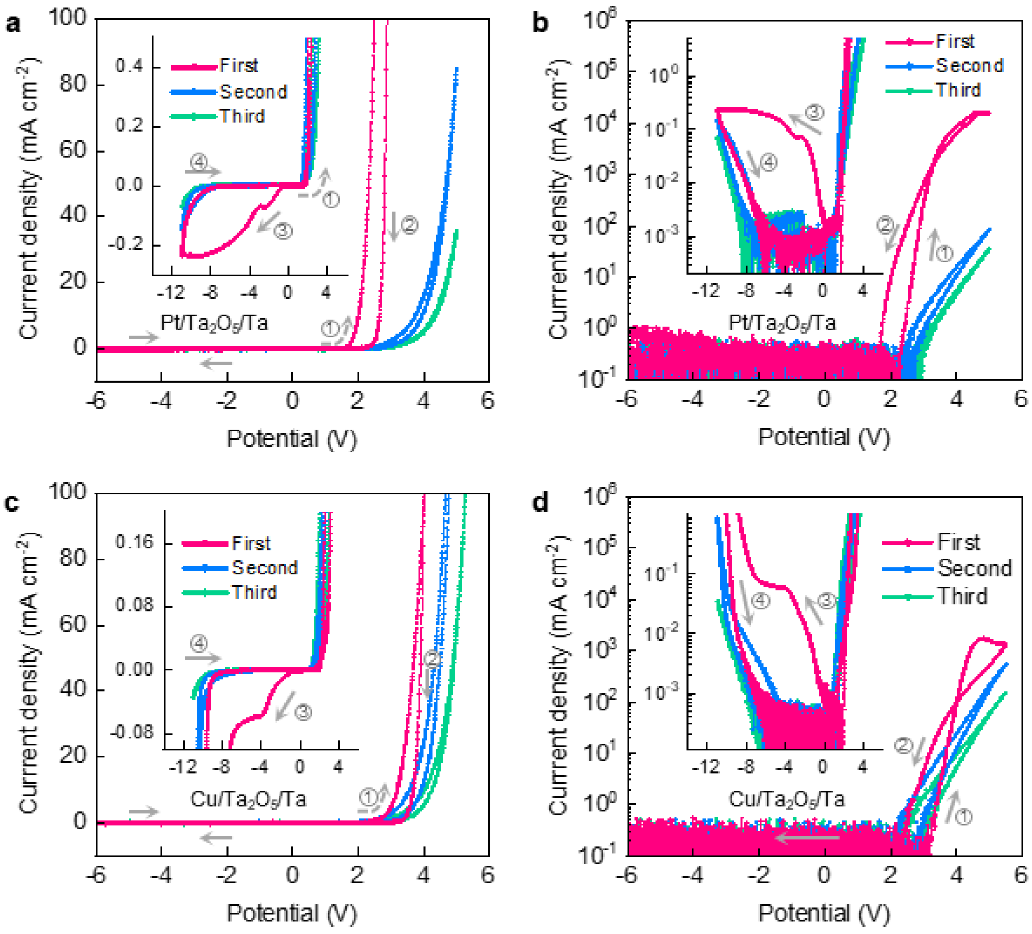

Supplementary Fig. 16. Diode-like electrical properties. a, Cyclic voltammograms Pt/Ta_2O_5/Ta and **c**, Cu/Ta_2O_5/Ta devices. **b** and **d** show the CVs on a logarithmic scale. The insets show high-revolution CV sweeps.

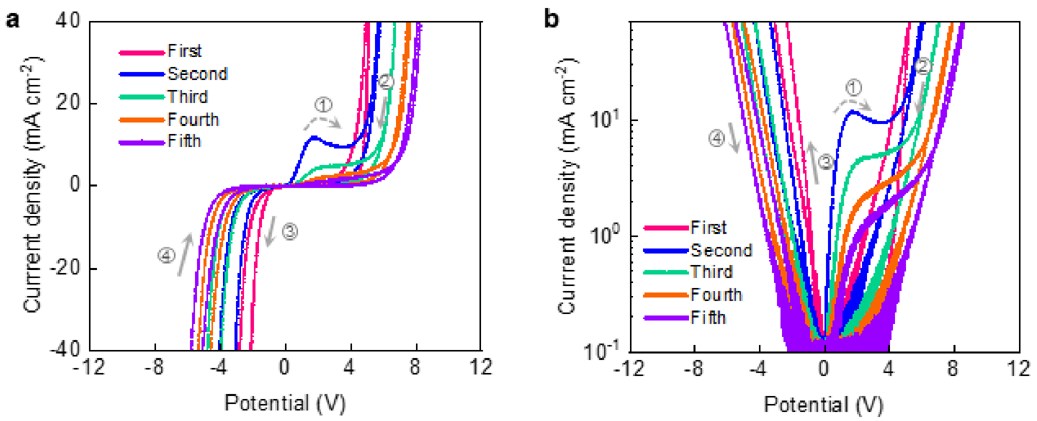

Supplementary Fig. 17. Cyclic voltammograms in Ta *vs*. Hf ohmic memristive systems. a, CVs when the electrical potentials were applied to Ta electrodes. **b**, CV presented in semilogarithmic scale. Here, the oxidation current density peaks are more pronounced, compared to Hf *vs*. Ta, Ta *vs*, Ta and Zr *vs*. Ta (see Fig. 2f and Supplementary Fig. 18).

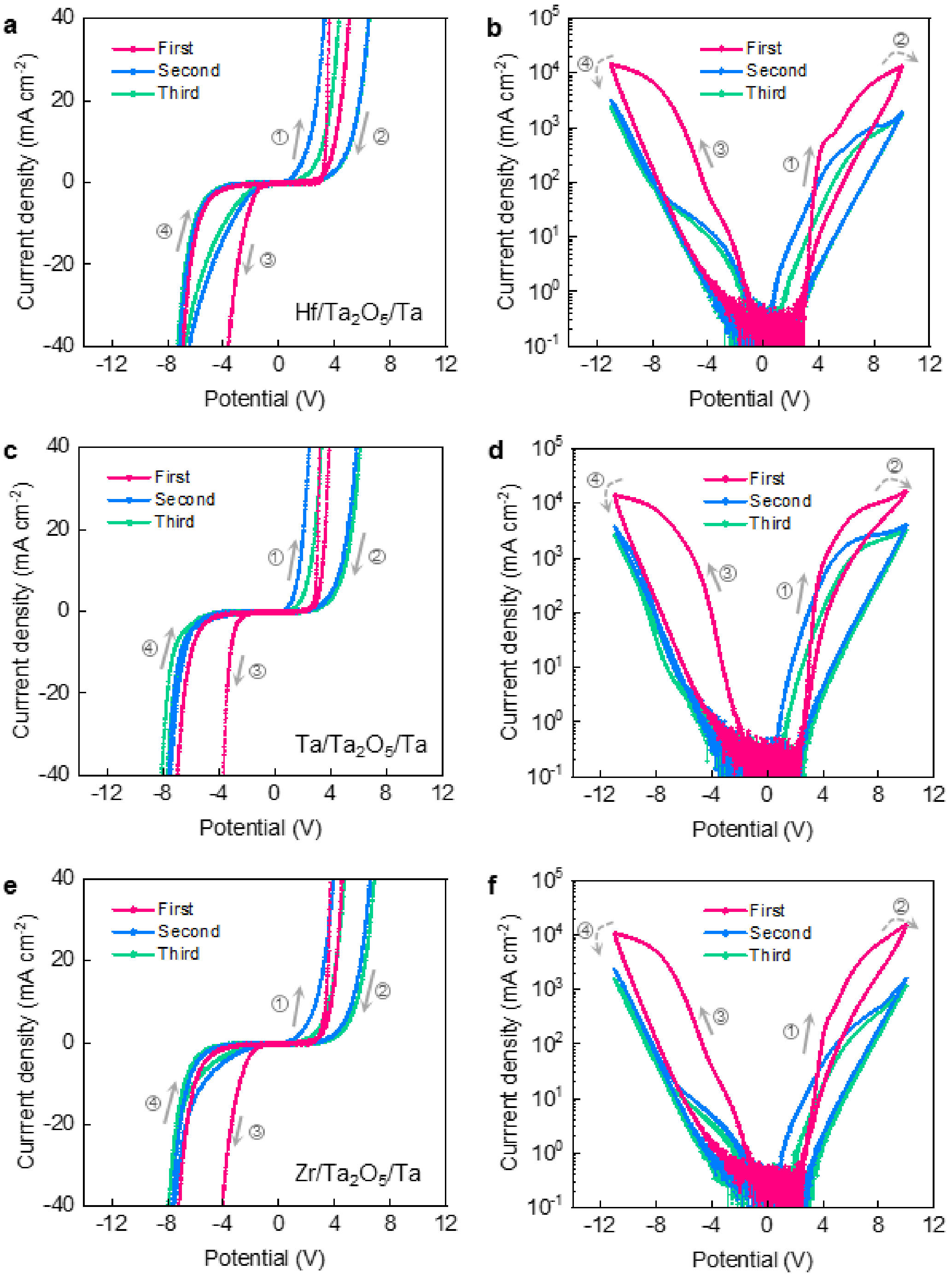

Supplementary Fig. 18. Cyclic voltammograms in ohmic memristive systems. a,**b**, Hf/Ta_2O_5/Ta. **c**,**d**,: Ta/Ta_2O_5/Ta. **e**,**f**,: Zr/Ta_2O_5/Ta. The CVs are presented in linear scale in a, b and c. In panels b, d and f the CVs are presented in semilogarithmic scale.

Supplementary Table 2: Standard electrode potentials. This table summarizes the standard electrode potentials E° (with respect to standard hydrogen electrode) of redox systems. The standard electrode potentials are taken from Ref.22.

**Half-cell reaction**	$\boldsymbol{E^{\circ}}$**/V**
Pt^{2+}/Pt [$Pt(OH)_2 + 2e^- \rightleftharpoons Pt + 2OH^-$]	0.14
Pt^{4+}/Pt [$PtO_2 + 4H^+ + 4e^- \rightleftharpoons Pt + 2H_2O$]	1.00
Pt^{4+}/Pt^{2+} [$PtO_2 + 2H^+ + 2e^- \rightleftharpoons PtO + H_2O$]	1.01
Pt^{2+}/Pt [$Pt^{2+} + 2e^- \rightleftharpoons Pt$]	1.18
Pt^{2+}/Pt [$PtOH^+ + H^+ + 2e^- \rightleftharpoons Pt + H_2O$]	1.2
Pt^{6+}/Pt^{4+} [$PtO_3 + 4H^+ + 2e^- \rightleftharpoons Pt(OH)_2^{2+} + H_2O$]	1.5
Pt^{6+}/Pt^{4+} [$PtO_3 + 2H^+ + 2e^- \rightleftharpoons PtO_2 + H_2O$]	1.7
Cu^{+}/Cu [$Cu_2O + H_2O + 2e^- \rightleftharpoons 2Cu + 2OH^-$]	–0.360
Cu^{2+}/Cu [$Cu(OH)_2 + 2e^- \rightleftharpoons Cu + 2OH^-$]	–0.222
Cu^{2+}/Cu^{+} [$2Cu(OH)_2 + 2e^- \rightleftharpoons Cu_2O + 2OH^- + H_2O$]	–0.080
Cu^{2+}/Cu^{+} [$Cu^{2+} + e^- \rightleftharpoons Cu^+$]	0.153
Cu^{2+}/Cu [$Cu^{2+} + 2e^- \rightleftharpoons Cu$]	0.3419
Cu^{+}/Cu [$Cu^{+} + e^- \rightleftharpoons Cu$]	0.521
Cu^{3+}/Cu^{2+} [$Cu_2O_3 + 6H^+ + 2e^- \rightleftharpoons 2Cu^{2+} + 3H_2O$]	2.0
Cu^{3+}/Cu^{2+} [$Cu^{3+} + e^- \rightleftharpoons Cu^{2+}$]	2.4
Hf^{4+}/Hf [$Hf^{4+} + 4e^- \rightleftharpoons Hf$]	–1.55
Hf^{4+}/Hf [$HfO_2 + 4H^+ + 4e^- \rightleftharpoons Hf + 2H_2O$]	–1.505
Hf^{4+}/Hf [$HfO^{2+} + 2H^+ + 4e^- \rightleftharpoons Hf + H_2O$]	–1.724
Hf^{4+}/Hf [$HfO(OH)_2 + H_2O + 4e^- \rightleftharpoons Hf + 4OH^-$]	–2.50
Ta^{3+}/Ta [$Ta^{3+} + 3e^- \rightleftharpoons Ta$]	–0.6
Ta^{5+}/Ta [$Ta_2O_5 + 10H^+ + 10e^- \rightleftharpoons 2Ta + 5H_2O$]	–0.75
Zr^{4+}/Zr [$Zr^{4+} + 4e^- \rightleftharpoons Zr$]	–1.45
Zr^{4+}/Zr [$ZrO_2 + 4H^+ + 4e^- \rightleftharpoons Zr + 2H_2O$]	–1.553
Zr^{4+}/Zr [$ZrO(OH)_2 + H_2O + 4e^- \rightleftharpoons Zr + 4OH^-$]	–2.36

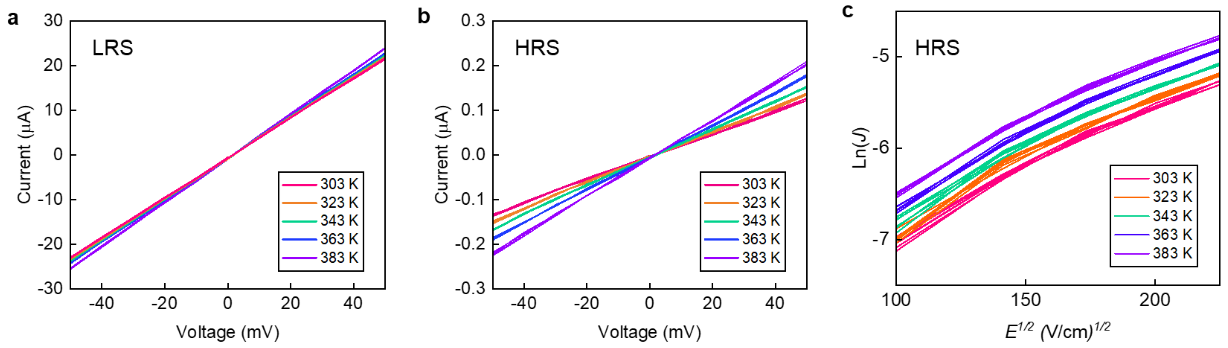

Supplementary Fig. 19. Temperature-dependent electrical characteristics in ohmic memristive devices. a, LRS currents show linear dependence on the voltage under various temperatures. **b**, HRS currents show linear dependence on the voltage under various temperatures. **c**, Fitting of HRS current using Schottky-emission model, the nonlinear curves indicate the current transport does not follow Schottky-emission mechanism. The voltage sweep range is between -50 mV to 50 mV, same as that in panels **a** and **b**.

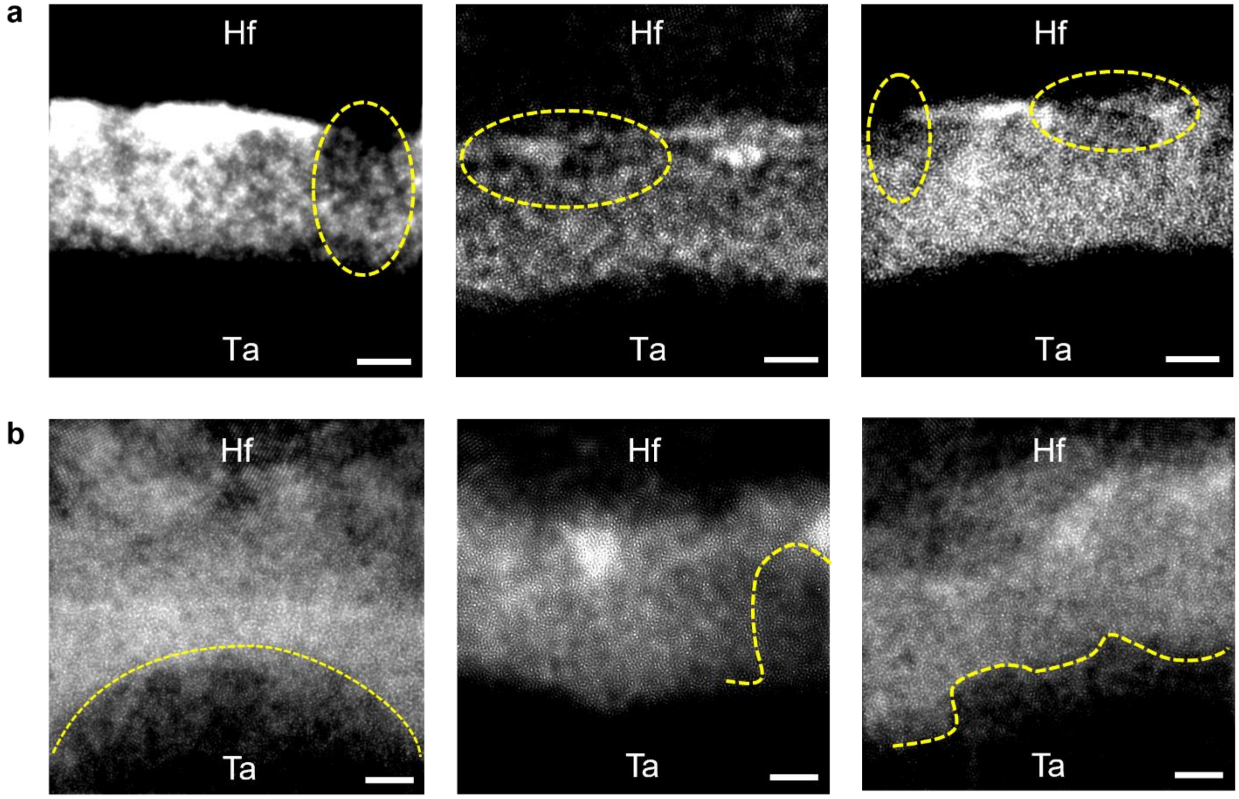

Supplementary Fig. 20. Additional TEM image showing the filament formation. a, Formation of the Ta conduction channel at the cathode (Hf) interface. The conduction channel grows toward the anode (Ta) under the applied electrical potential. Scale bar, 4 nm. **b**, Formation of the O-deficient (Ta-rich) clusters when the top Hf electrode was positively biased. The ohmic electrode Hf scavenges oxygen (O^{2-}) from the oxide and the IrO_2 layer (see Fig. 3k and Supplementary Fig. 23), generating oxygen vacancies at the cathode (Ta) interface. Scale bar, 2 nm.

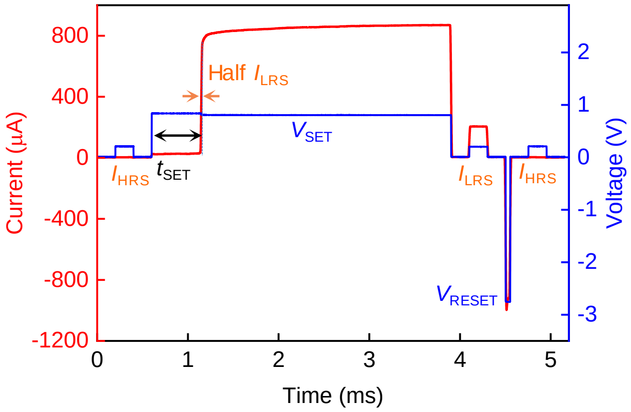

Supplementary Fig. 21. SET kinetics measurement. This figure shows temporal current response under applied pulse voltage. The t_{SET} (width of the black arrow) is defined as the time difference between the half of the SET pulse rising edge and the half of the SET current rising edge.

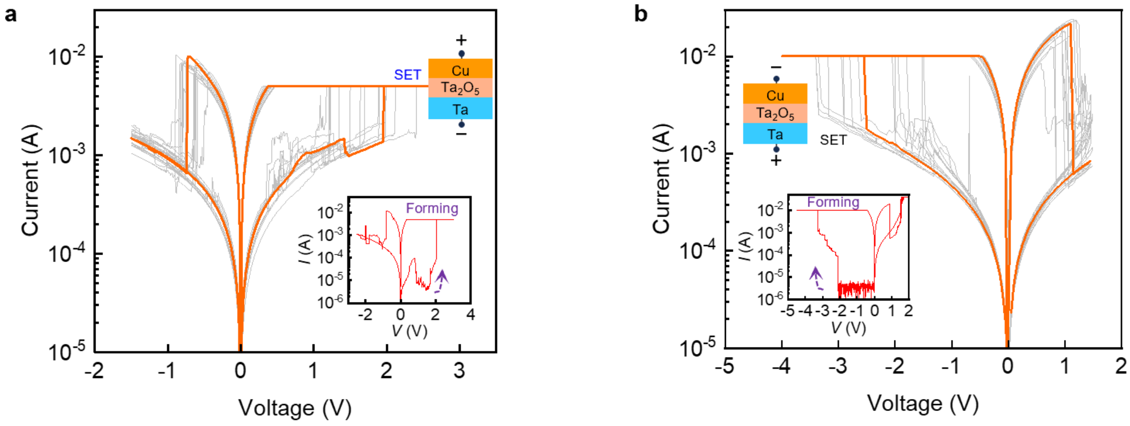

Supplementary Fig. 22. Resistive switching in Cu/Ta_2O_5/Ta based memristive device. a, Typical ECM-type resistive switching when using Cu as active electrode. The forming and SET operations were realized by applying positive voltages to Cu electrode. Inset: forming I-V curve **b**, ECM-type resistive switching observed when a negative voltage was applied to Cu (positive to Ta) in forming and SET operations. Inset: forming *I*-*V* curve.

Supplementary Table 3: Comparison of working mechanisms between ECM, VCM and ohmic (FCM) memristive devices. (Abbreviation: AE, active electrode; IE, inert electrode; OE, ohmic electrode; SE, Schottky electrode)

Device type	Structure	Mobile specie(s)	Filament type	Resistance change	LRS	HRS
ECM	AE/Oxide/IE<br><br>(e.g. Cu/SiO2/Pt	Metal cation	Metallic	Metallic filament formation/dissolution	Metallic filament	Filament dissolved completely
VCM	OE/Oxide/SE<br><br>(e.g.Ta/Ta_2O_5/Pt)	Oxygen anion (oxygen vacancies)	Reduced oxide<br><br>Consists of Plug and Disc	Shift of oxygen vacancies in/out of the Disc and related Schottky barrier height change	Reduced interfacial barrier height	Increased interfacial barrier height
FCM	OE/Oxide/OE (Ta/Ta_2O_5/Hf)	Metal cation, oxygen anion	Reduced oxide	Change of filament conductivity due oxidation and length change and thus, the resistance	Filament of reduced oxide	Filament (partially) oxidized and with reduced length

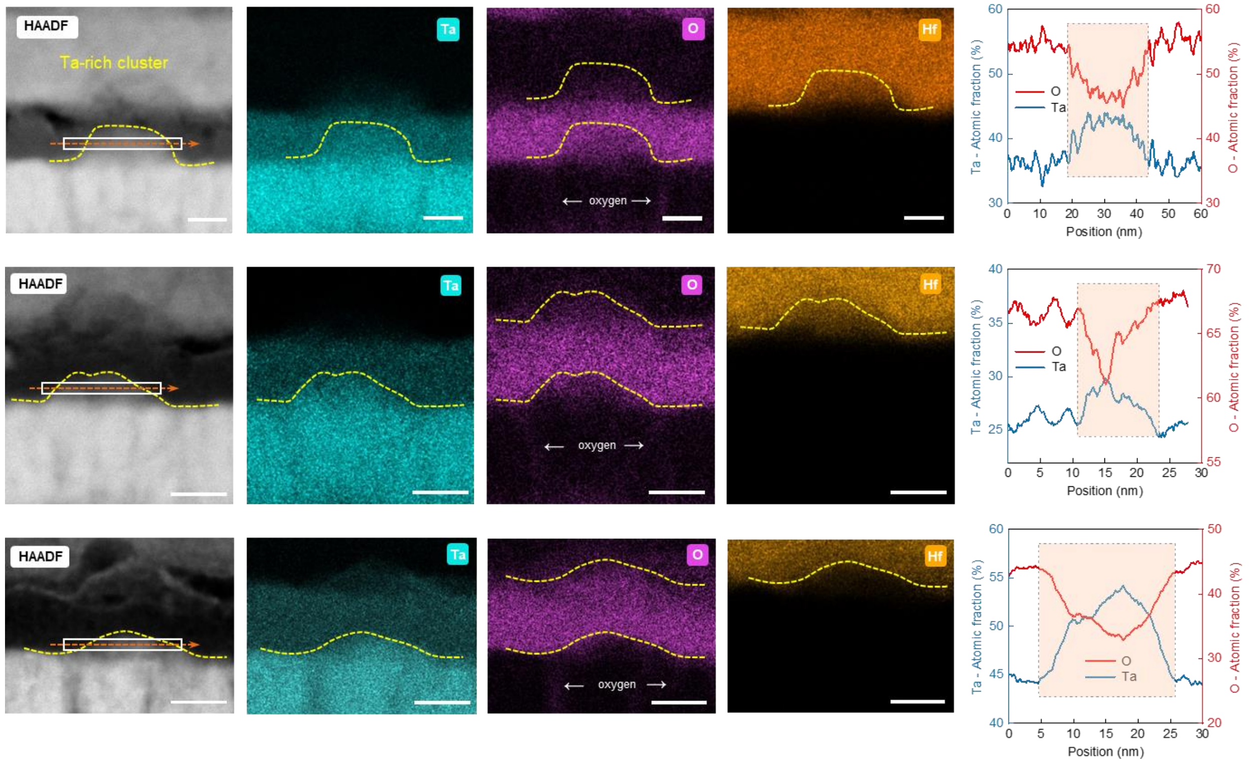

Supplementary Fig. 23. Additional cross-sectional TEM images and EDS profiling. The presented TEM images show the formation of O-deficient clusters at the Ta/Ta_2O_5 interface. The EDS line scan results shown on the right depict the concentration of the Ta and O elements. It can be seen that the cluster regions exhibit a substantial decrease of the O concentration. Scale bars, 10 nm.

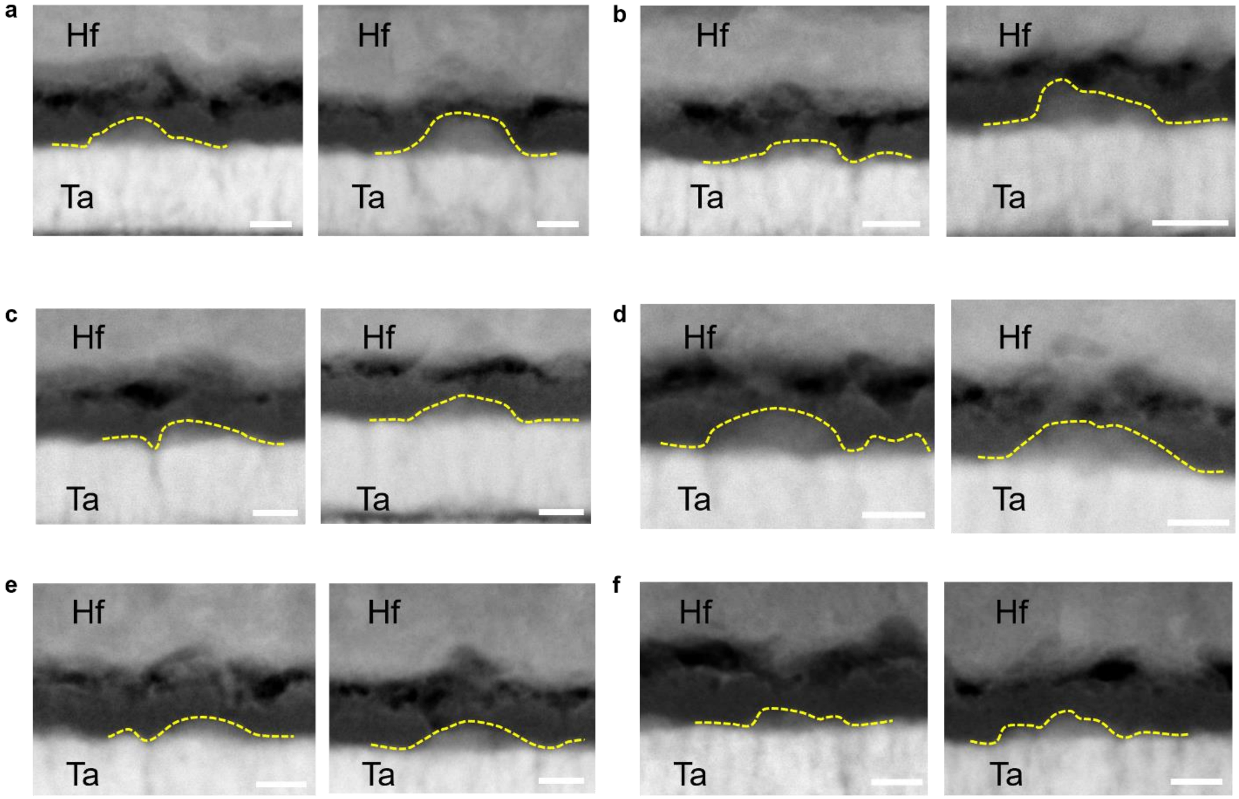

Supplementary Fig. 24. Multiple oxygen-deficient filaments within the oxide layer. Scale bar, 10 nm.

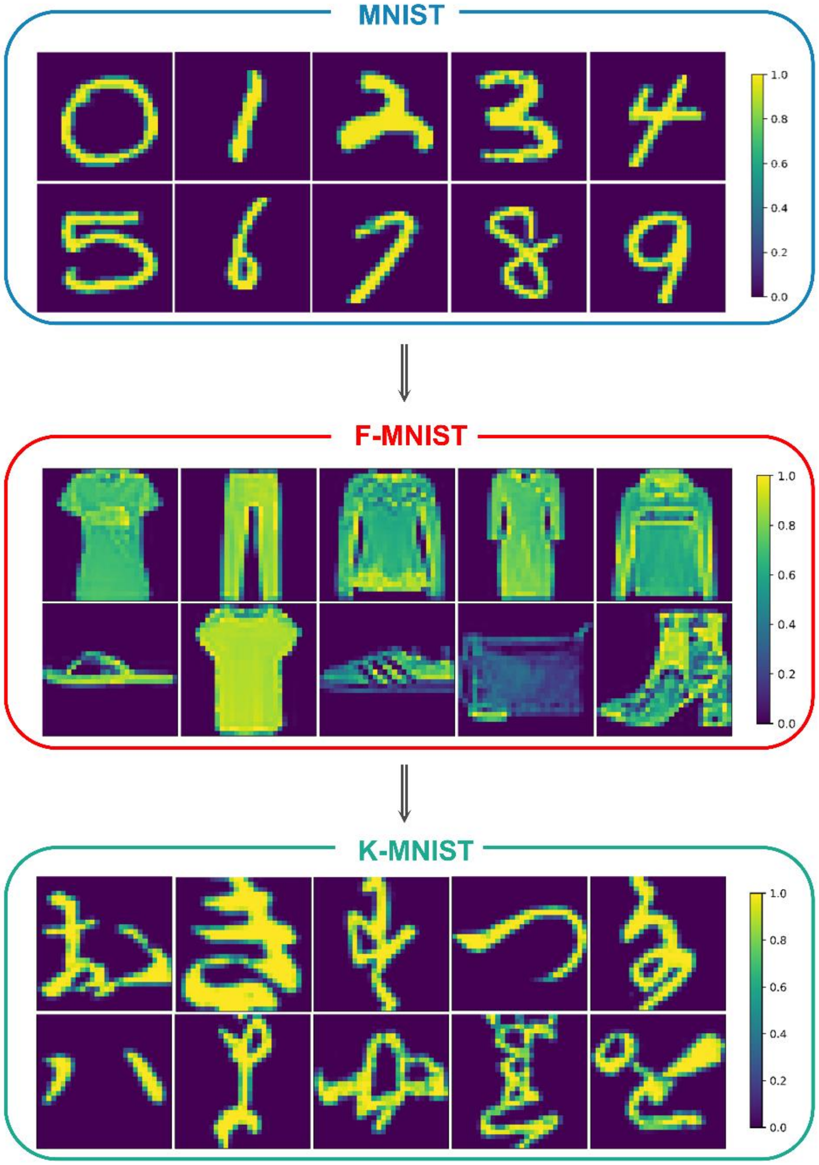

Supplementary Fig. 25. Three different tasks (MNIST[23], F-MNIST[24], K-MNIST[25]) for testing the neural-networks continual learning capacity.

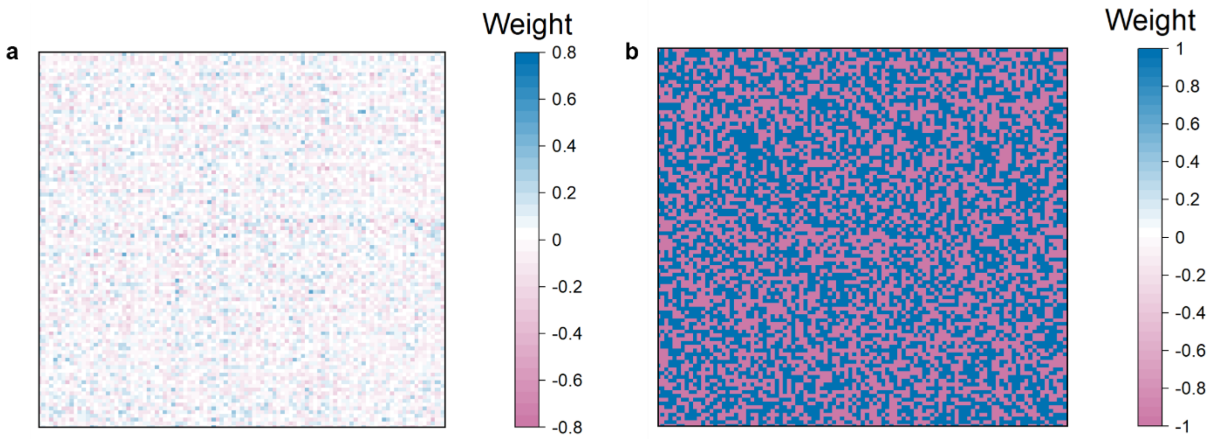

Supplementary Fig. 26. Weights mapping for part of the hidden layer, including the float-point and binary-value format.

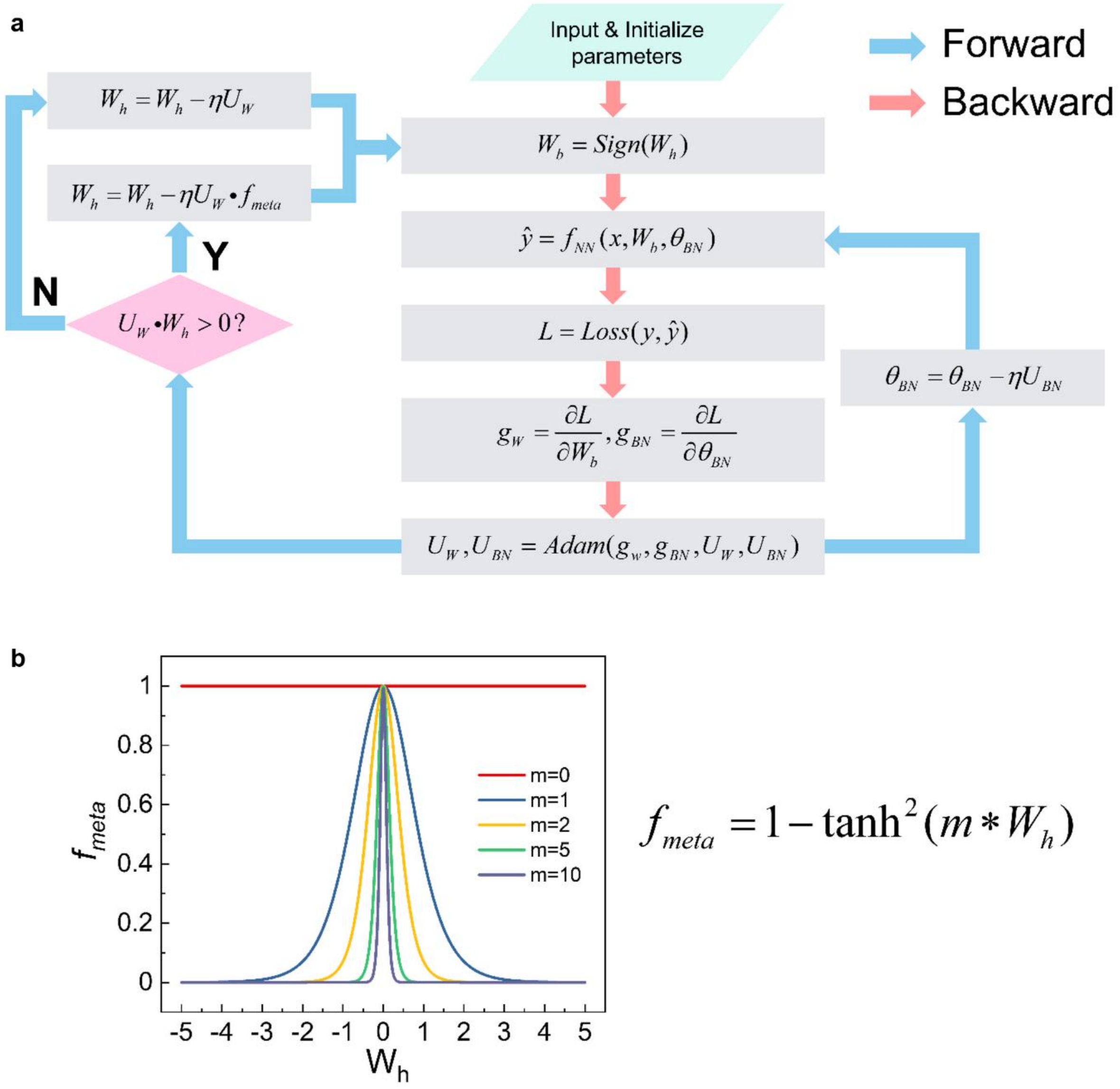

Supplementary Fig. 27. Calculation process for the metaplasticity-inspired neural network. **a**, Schematic of the forward and backward procedure in the neural network computation process, where the binary-value weights are adopted for inference and the analog-value hidden weights are updated with a nonlinear function and gradients. **b**, The nonlinear function f_{meta} whose variables include hyperparameters m and analog-value weights W_h. f_{meta} can be written as: $\boldsymbol{f_{meta} = 1 - \tanh(m \times W_h)}$

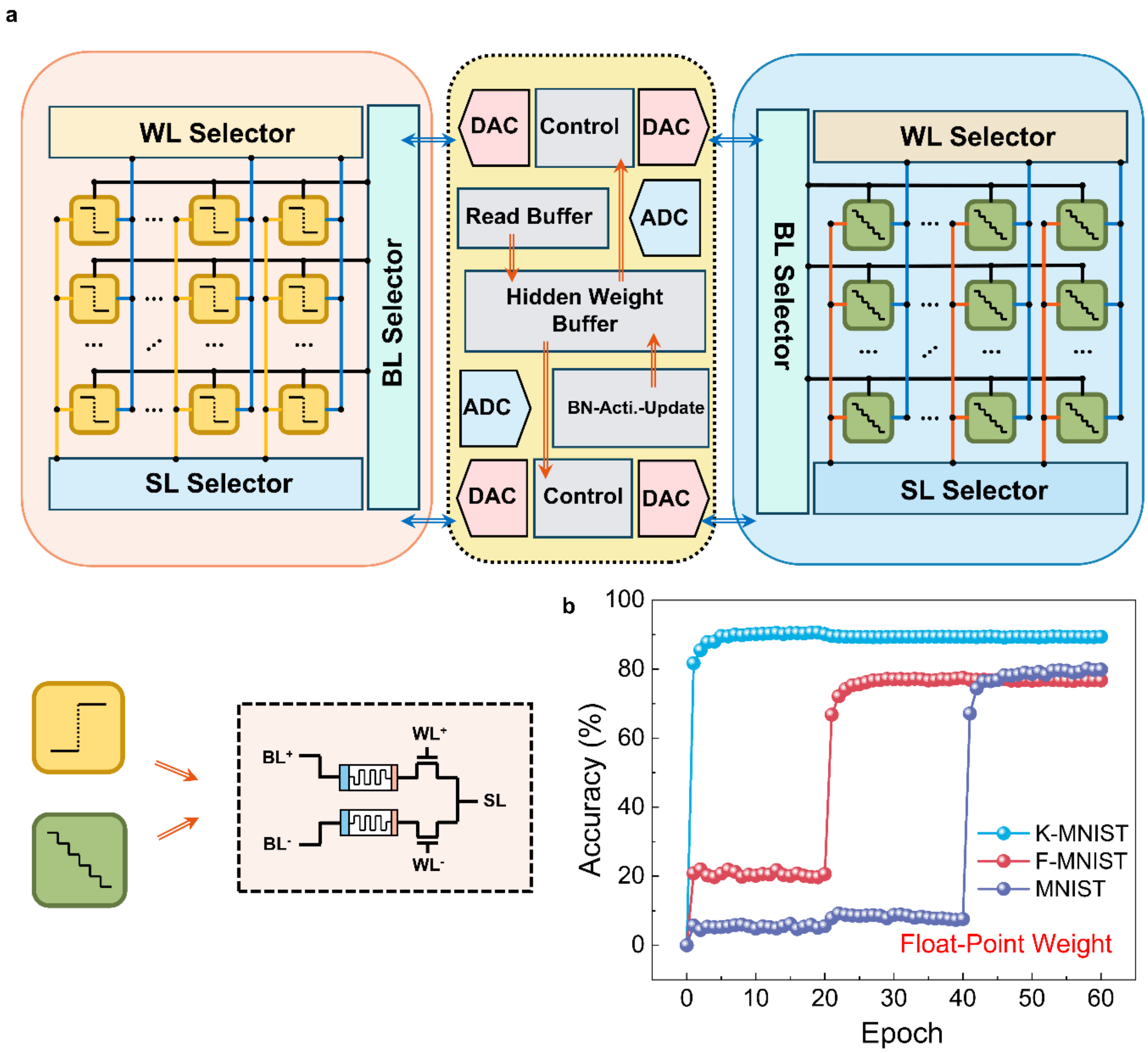

Supplementary Fig. 28. Hardware architecture for implementing metaplasticity-inspired continual learning and digital baseline performances of the continual learning. a, The fully-connected neural network structure was kept as 784-500-200-10, in which the numbers of neurons in the two hidden layers were set as 500 and 200, respectively, due to the size of datasets, the numbers of neurons in the input layer and output layer were fixed at 784 and 10, respectively. There are two kinds of memristor arrays, which are both based on the novel ohmic memristors. To represent the positive and negative weight values, the differential structure is employed, in which two devices correspond to one weight in the neural network. Considering the time-consuming programming procedures of analog weights, the hidden buffer module is set for storing the value of the hidden weights temporarily, once the training processes for the new tasks are finished, the analog weight module will get programmed from the hidden weight buffer. **b,** Network performances with the digital float-point weights used for training, in which all the weights are implemented at the software and other neural parameters are kept the same with the following simulated hardware-implemented neural network.

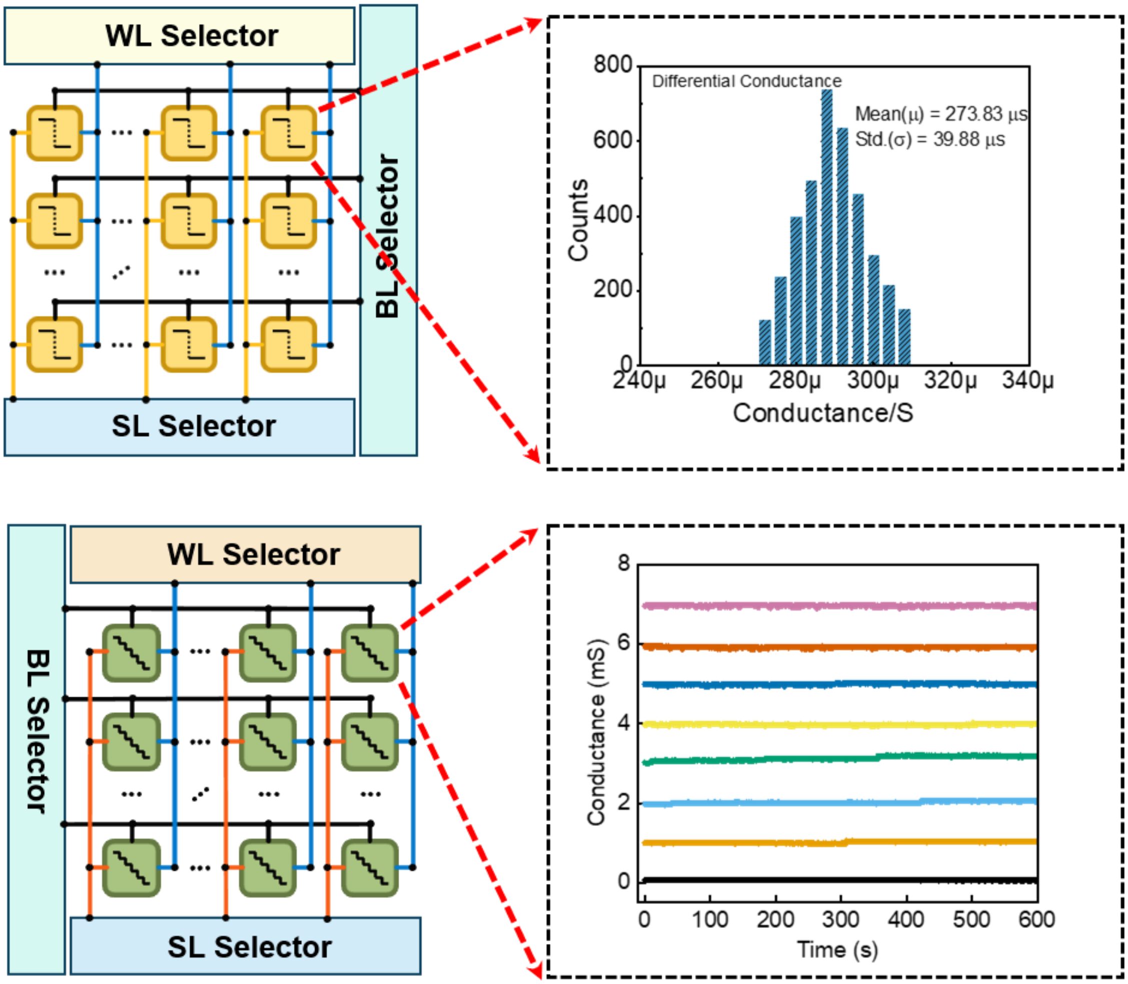

W_{real} samples from: $N(273.8\mu S, (39.88\mu S)^2)$. $W_{real\text{-}model} = W_{real} \cdot \lambda_{real}$

$X_{out} = X_{in} \times W_{real\text{-}model}$

W_{hidden} samples from: $U(W_{min}, W_{max})$, under limited states. $W_{hidden\text{-}model} = W_{hidden} \cdot \lambda_{hidden}$

$W_{hidden\text{-}model}^{n+1} = W_{hidden\text{-}model}^{n} + lr \cdot Gradients$

Supplementary Fig. 29. The quantized details between the devices' conductances and neural network's weight values. For taking the measured devices' weights into the neural network model, the real weights samples from $N(273.8\mu S,(39.88\mu S)^2)$, which is the statistics in Supplementary Fig. 6. The hidden weights samples from $U(W_{min}, W_{max})$, the uniform distribution is controlled by the feedback programming method. Both for the real weights and hidden weights, the linear scale factor λ is adopted to better map the real device conductance into model weights, and the linear mapping of hardware parameters can ensure no difference in the final neural network results.

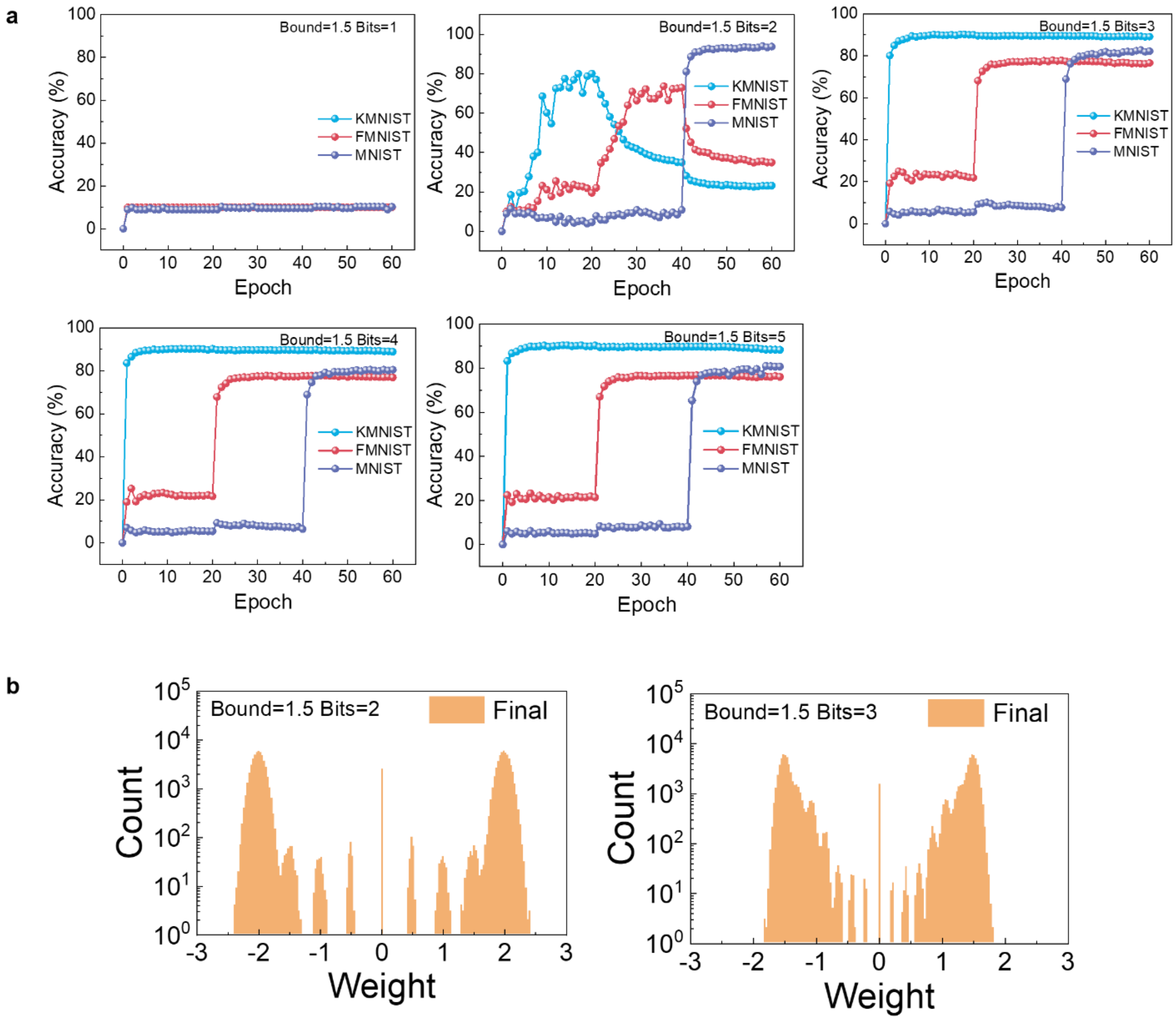

Supplementary Fig. 30 Network performance for different quantized bits precision within the same quantized bounds. **a**, The quantized bound was chosen at 1.5. **b**, The final weights distribution of the output layer (500 × 200) for the 2-bit and 3-bit precision.

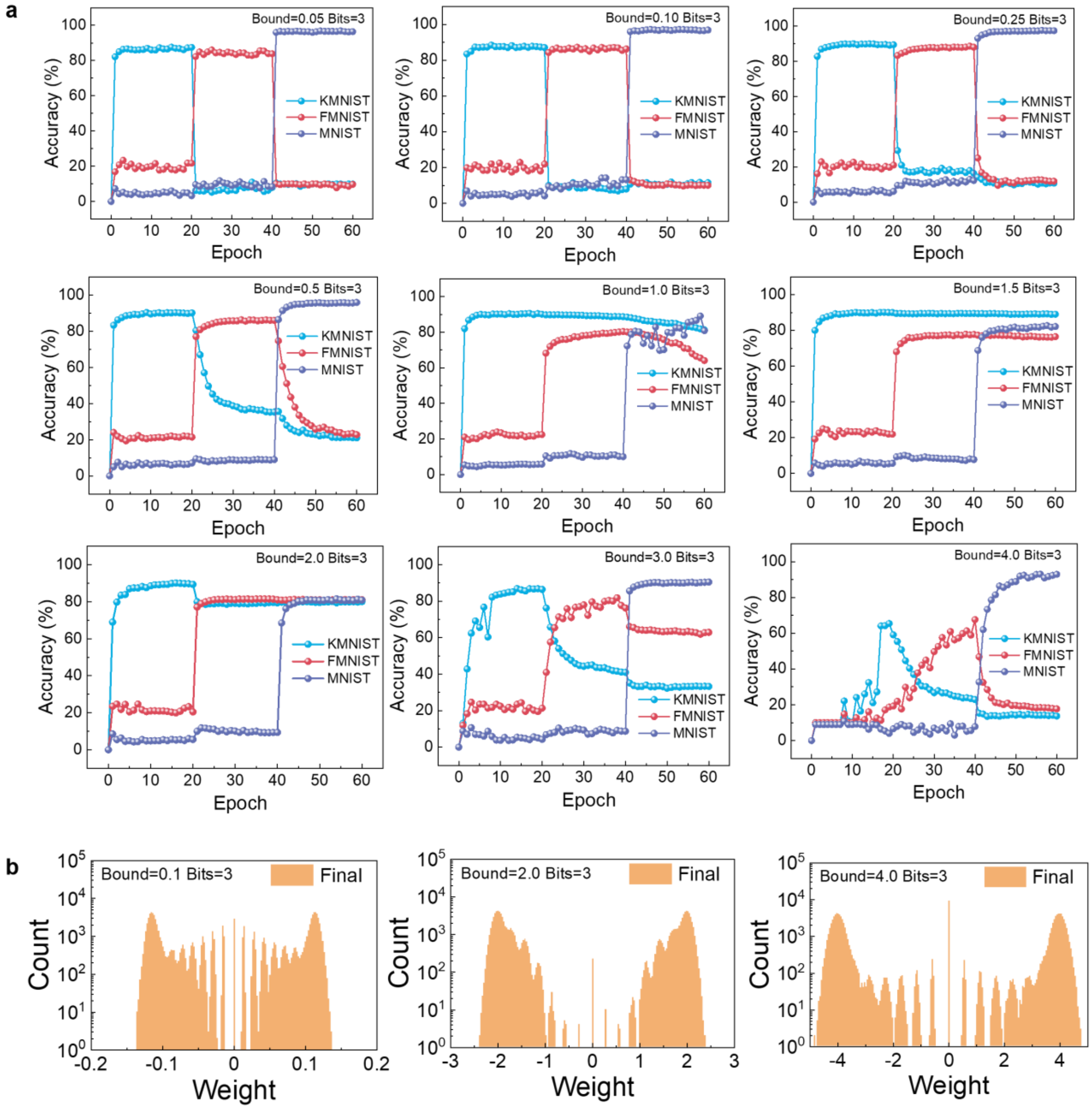

Supplementary Fig. 31. Network performances for different quantized bounds at the same quantized bits. a, The quantized bits were chosen at 3. **b**, The final weights distribution of the hidden layer (500×200) for the quantized bound of 0.1, 2.0 and 4.0, respectively.

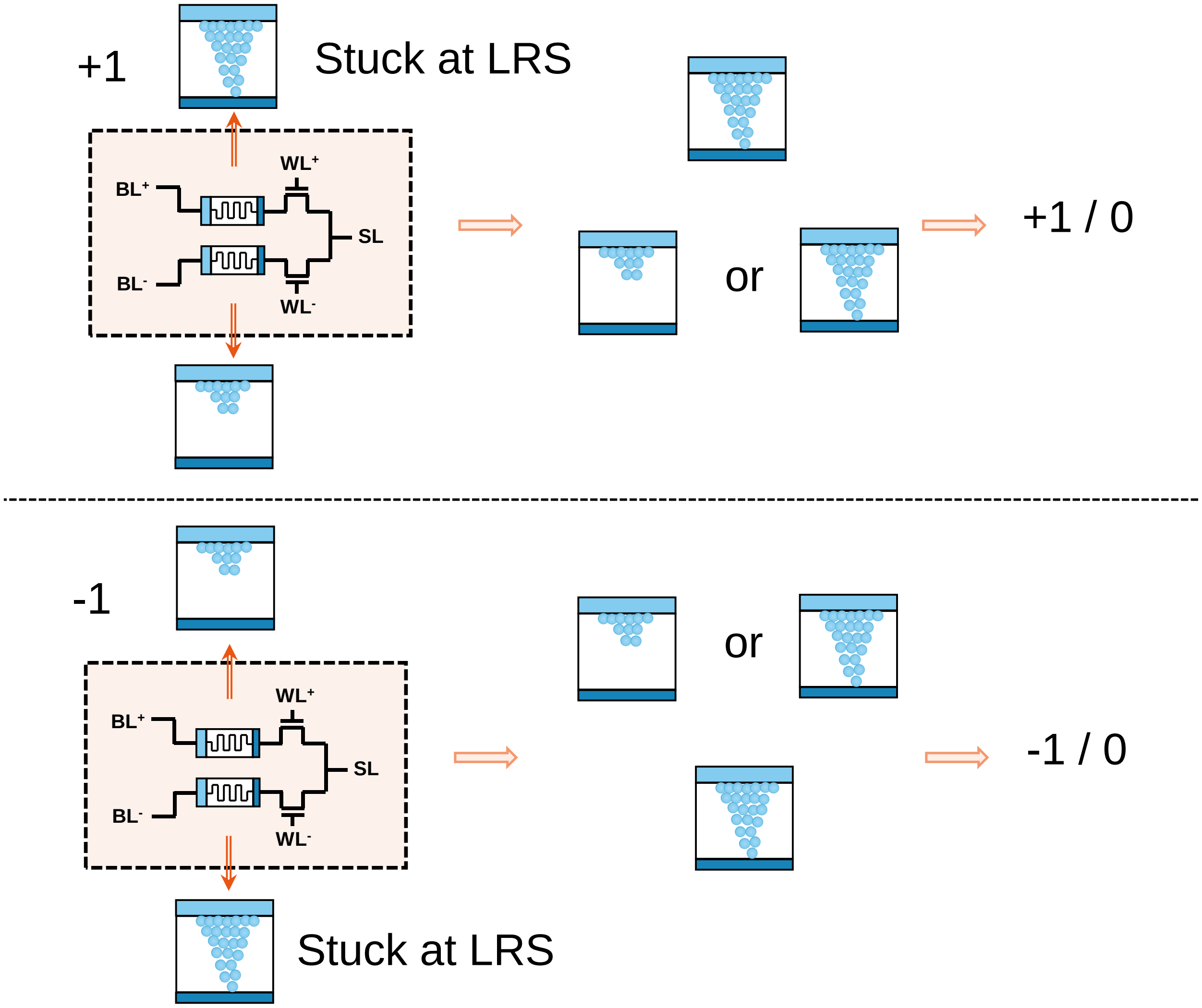

Supplementary Fig. 32. The schematic of impact on cell updating when device is stuck at LRS. In the conventional VCMs, such as $Ta/Ta_2O_5/Pt$ and $Hf/Ta_2O_5/Pt$, the device would be stuck at LRS after frequent programming operations, then the cell weight will be fixed at ±1 or 0. With the interface engineering, the ohmic memristor can better overcome this issue and is suitable for the continual learning application which requires more frequent network training and weights updating.

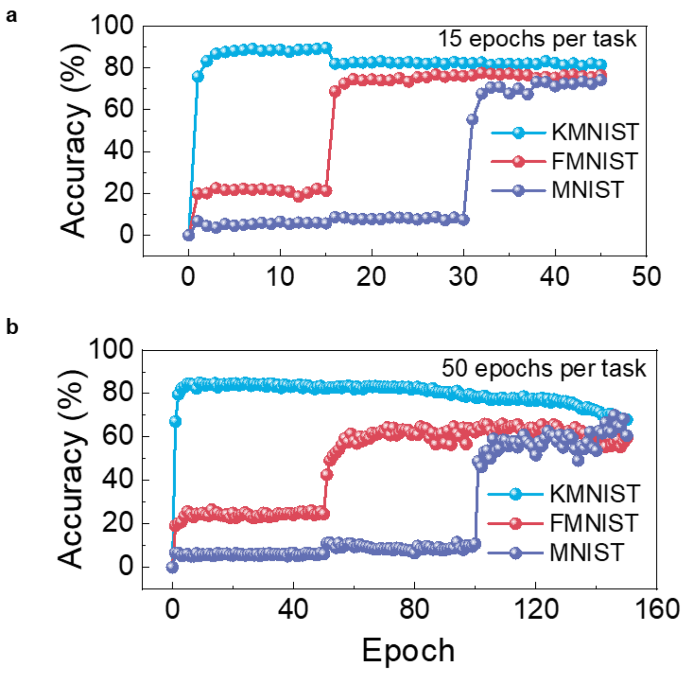

Supplementary Fig. 33. The schematic of impact on cell updating when device is stuck at LRS. Neural network performances for different epochs in training each task under the same failure ratio at 0.2%.

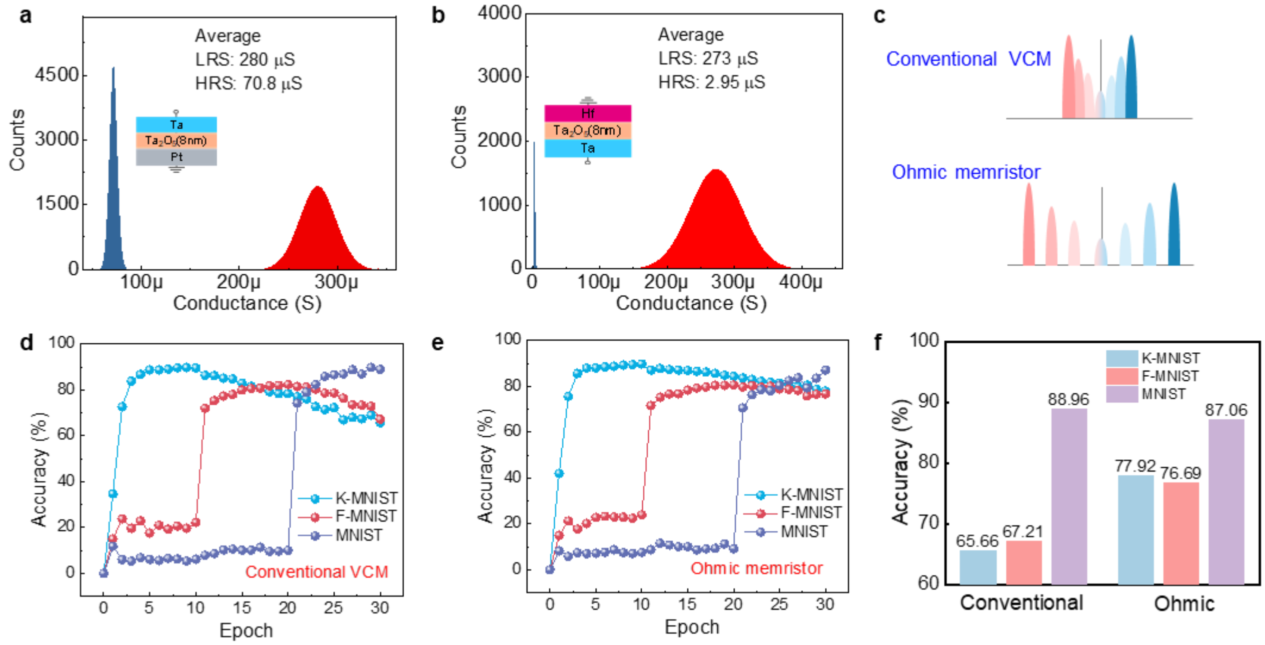

Supplementary Fig. 34. Comparison of neural networks performance. The schematic of impact on cell updating when device is stuck at LRS. The LRS and HRS distribution of conventional VCM (a) and ohmic memristor (b), in which the R_{OFF}/R_{ON} ratio of ohmic memristor is larger than that of conventional VCM. **c.** The schematic of the overlap among multi-level conductance states in conventional VCM and ohmic memristor devices. d,e, Neural network performances for the conventional VCM and ohmic memristor under the same programming errors. f, The comparison of the final accuracies of different tasks for the conventional VCM and ohmic memristor.

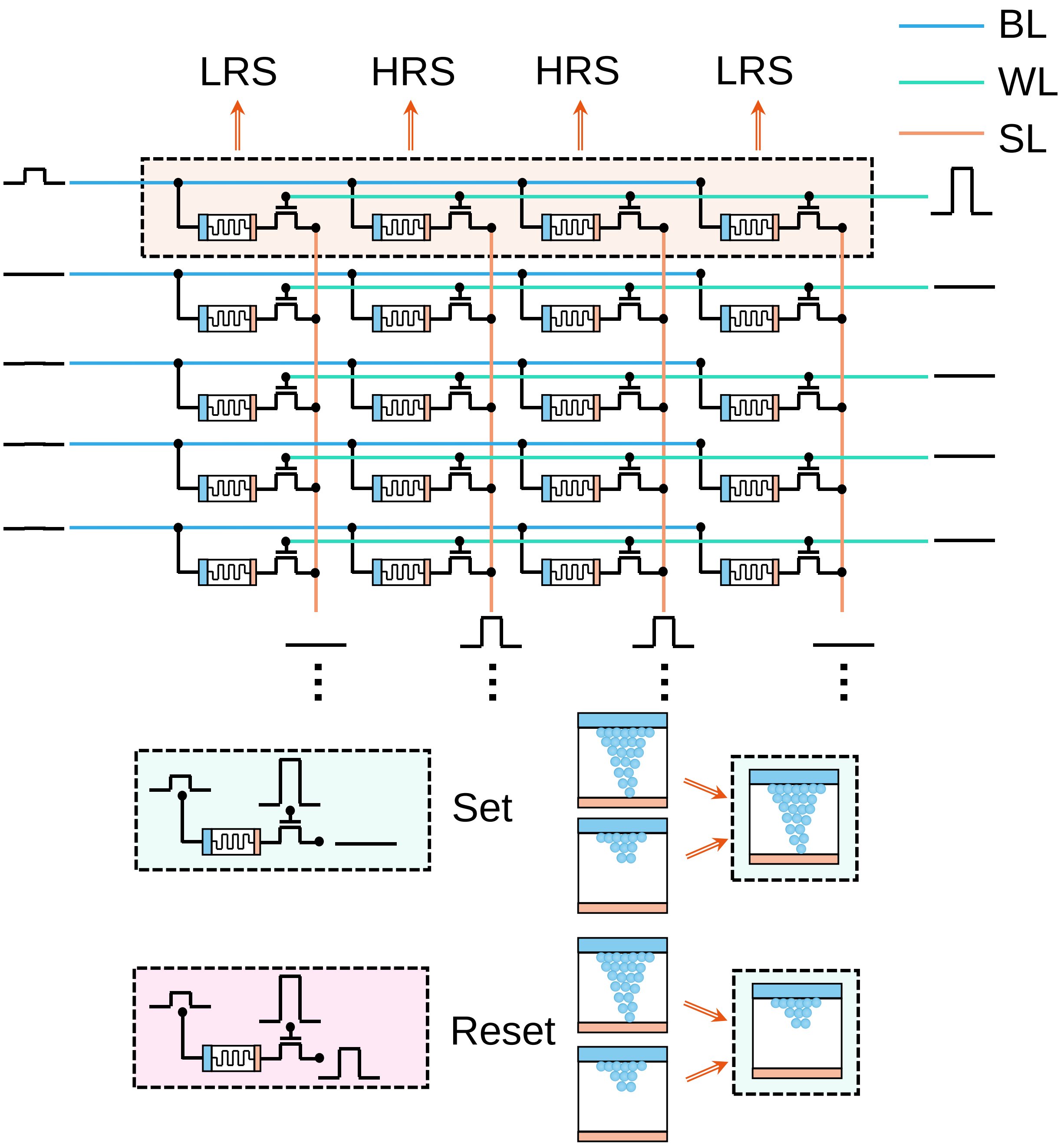

Supplementary Fig. 35. The schematic of the parallel programming method for devices' binary switching. To accelerate the binary programming, we employ the parallel programming method, in which a train of designed pulses are input from SL while the corresponding BL is set at a certain level and WL is set at V_{DD}, the different polarity of voltage drop on each memristor cell will enable the device to set or reset. The parallel programming method ensures that device states only depend on the current waveforms and have no relation with previous states.

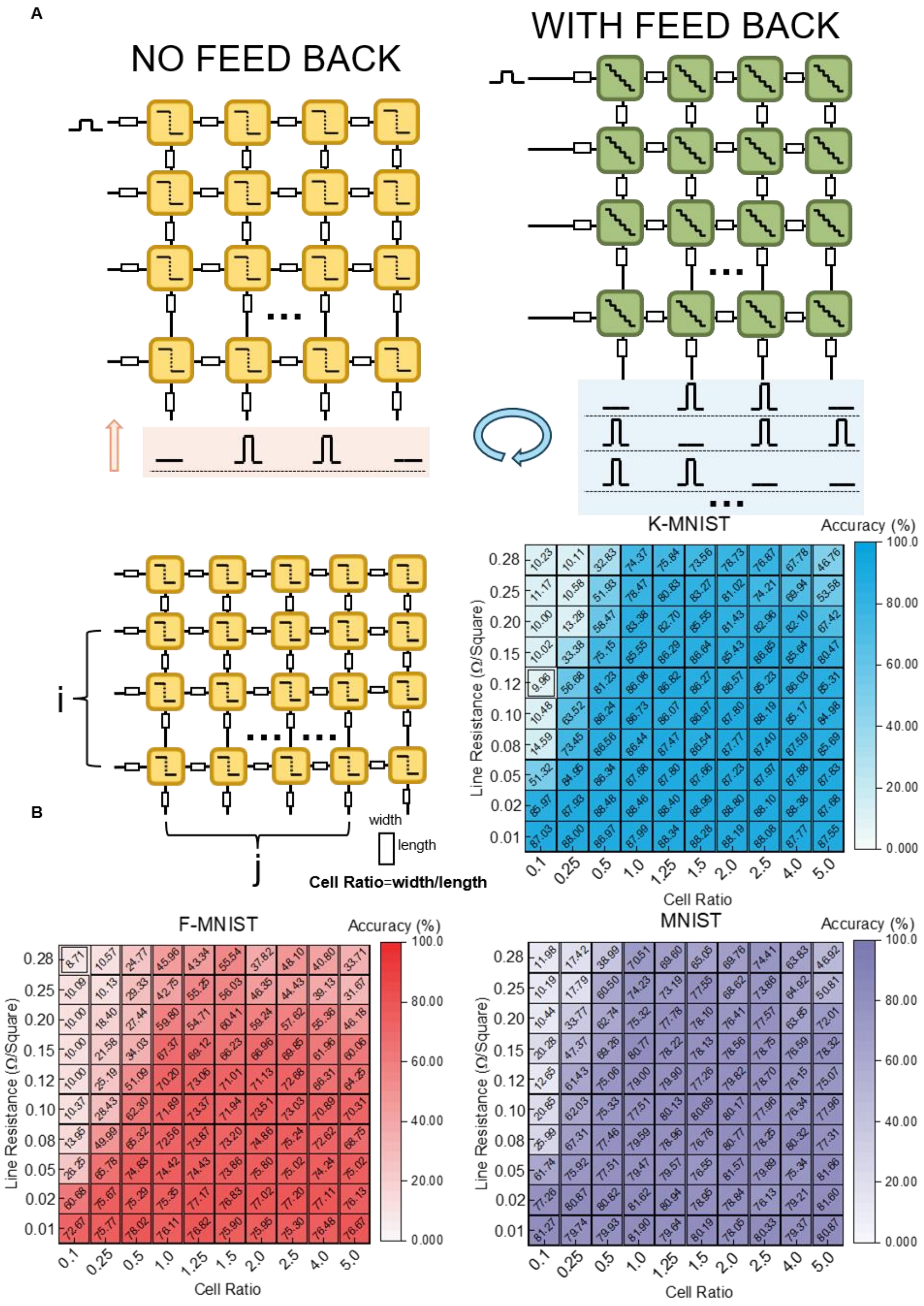

Supplementary Fig. 36. The effect of different line resistances and cell ratios on neural network performances. a, The schematic for programming binary weights and analog weights, in which the feedback write-verify method is implemented in analog programming, the final analog weight values include the line resistance, while the binary weight switching without feedback needs to be considered the effect of line resistances. **b**, The final accuracies of three tasks under different line resistances and cell ratios.

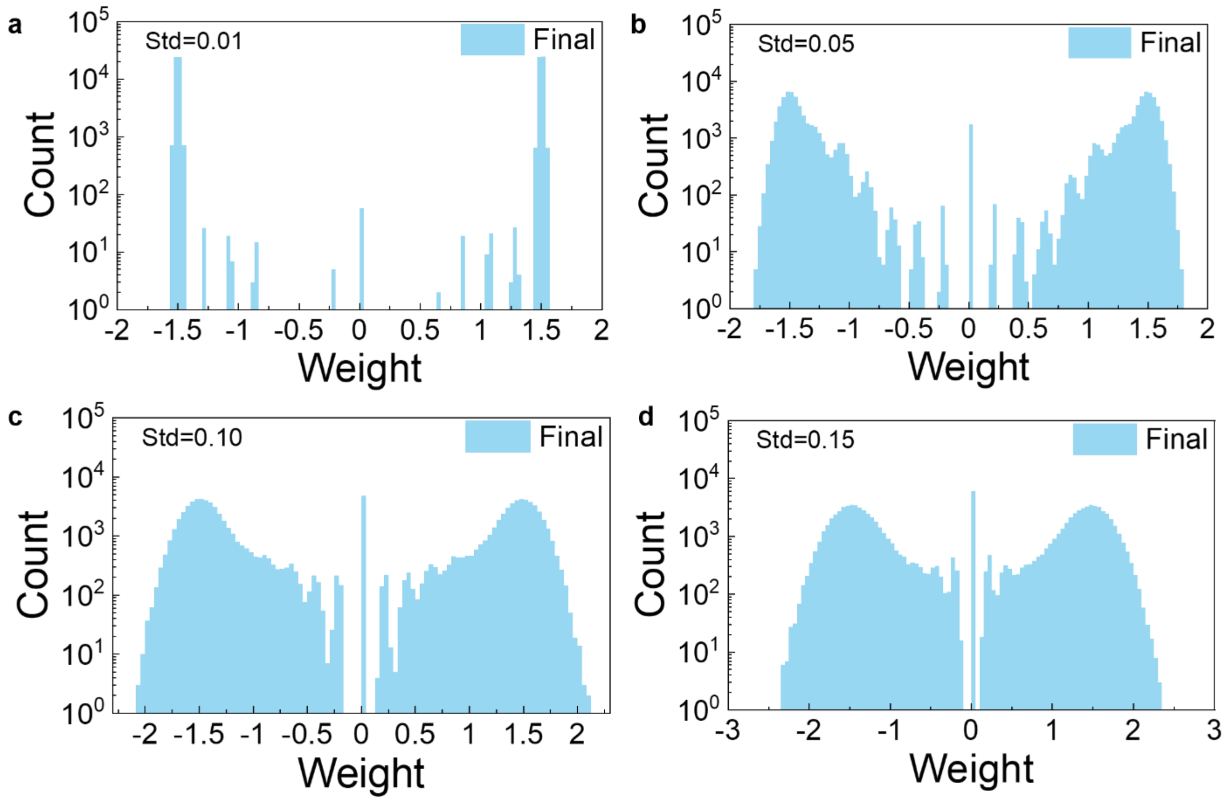

Supplementary Fig. 37. The final weight distributions of the hidden layer (500×200) for the different programming errors at the standard variation of 0.01**(a)**, 0.05**(b)**, 0.10**(c)** and 0.15**(d)**, respectively.

Supplementary Table 4: Summary of electrode and metal oxide deposition parameters. All deposition processes were conducted at room temperatures.

**RF Magnetron Sputtering (Cluster Tool 1000)**					
Target (material)	Sputter power (W)	Pressure (μbar)	Ar flux (sccm)	O_2 flux (sccm)	Rate (nm min^{-1})
Hf	60	5	15	–	~20
HfO_2	13	10	15	–	~0.95
Ir (IrO_2)	30	3	20	2	~18.3
Pt	80	5	15	–	~30
Ta	25	4	15	–	~5.5
Ta (Ta_2O_5)	100	40	12	8	~1.8
Zr	60	5	15	–	~15

**DC Magnetron Sputtering (CS 500ES)**					
Target (material)	Sputter power (W)	Pressure (μbar)	Ar flux (sccm)	N_2 flux (sccm)	Rate (nm min^{-1})
Pt	200	5.5	30	–	~69
Ti (TiN)	200	1.1	27	3	~7.4

**Electron beam evaporation (CS 500ES)**				
Target	Voltage (kV)	Current (A)	Pressure (μbar)	Rate (nm s^{-1})
Cu	~8.43	~0.2	1.2	0.01

References

[1] Prakash, A., Jana, D., Samanta, S. & Maikap, S. Self-compliance-improved resistive switching using Ir/TaOx/W cross-point memory. Nanoscale Res Lett 8, 527 (2013).

[2] Akbari, M., Kim, M.-K., Kim, D. & Lee, J.-S. Reproducible and reliable resistive switching behaviors of AlO X /HfO X bilayer structures with Al electrode by atomic layer deposition. RSC Advances 7, 16704–16708 (2017).

[3] Berthaud, F. et al. In-Depth Analysis of Transistor Influence on OxRAM Performance in Memory Bitcell, With Technology Scaling Perspectives. IEEE Trans. Electron Devices 71, 2721–2728 (2024).

[4] Shin, D. H. et al. Multiphase Reset Induced Reliable Dual-Mode Resistance Switching of the Ta/HfO2/RuO2 Memristor. ACS Appl. Mater. Interfaces 16, 13, 16462–16473 (2024).

[5] Chen, Z. et al. High-performance HfO x /AlO y -based resistive switching memory cross-point array fabricated by atomic layer deposition. Nanoscale Res Lett 10, 70 (2015).

[6] Munjal, S. & Khare, N. Valence Change Bipolar Resistive Switching Accompanied With Magnetization Switching in CoFe2O4 Thin Film. Sci. Rep. 7, 12427 (2017).

[7] Beckmann, K., Holt, J., Manem, H., Van Nostrand, J. & Cady, N. C. Nanoscale Hafnium Oxide RRAM Devices Exhibit Pulse Dependent Behavior and Multi-level Resistance Capability. MRS Adv. 1, 3355–3360 (2016).

[8] Chen, Y.-S. et al. Good Endurance and Memory Window for Ti/HfOx Pillar RRAM at 50-nm Scale by Optimal Encapsulation Layer. IEEE Electron Device Lett. 32, 390–392 (2011).

[9] Chakrabarti, B., Galatage, R. V. & Vogel, E. M. Multilevel Switching in Forming-Free Resistive Memory Devices With Atomic Layer Deposited HfTiOx Nanolaminate. IEEE Electron Device Lett. 34, 867–869 (2013).

[10] Mahata, C., Kang, M. & Kim, S. Multi-Level Analog Resistive Switching Characteristics in Tri-Layer HfO2/Al2O3/HfO2 Based Memristor on ITO Electrode. Nanomaterials 10, 2069 (2020).

[11] Kim, W. et al. Forming-free metal-oxide ReRAM by oxygen ion implantation process. in 2016 IEEE International Electron Devices Meeting (IEDM) 4.4.1-4.4.4 (2016).

[12] Lee, M.-J. et al. A fast, high-endurance and scalable non-volatile memory device made from asymmetric Ta2O5−x/TaO2−x bilayer structures. Nature Mater 10, 625–630 (2011).

[13] Ismail, M., Mahata, C. & Kim, S. Forming-free Pt/Al2O3/HfO2/HfAlOx/TiN memristor with controllable multilevel resistive switching and neuromorphic characteristics for artificial synapse. Journal of Alloys and Compounds 892, 162141 (2022).

[14] González, M. B. et al. Synaptic devices based on HfO2 memristors. in Mem-elements for Neuromorphic Circuits with Artificial Intelligence Applications (eds. Volos, C. & Pham, V.-T.) 383–426 (Academic Press, 2021). doi:10.1016/B978-0-12-821184-7.00028-1.

[15] Ahn, M. et al. Memristors Based on (Zr, Hf, Nb, Ta, Mo, W) High-Entropy Oxides. Adv. Electron. Mater. 7, 2001258 (2021).

[16] Ismail, M. et al. Improved Endurance and Resistive Switching Stability in Ceria Thin Films Due to Charge Transfer Ability of Al Dopant. ACS Appl. Mater. Interfaces 8, 6127–6136 (2016).

[17] Mikhaylov, A. N. et al. Bipolar resistive switching and charge transport in silicon oxide memristor. Materials Science and Engineering: B 194, 48–54 (2015).

[18] von Witzleben, M. et al. Study of the SET switching event of VCM-based memories on a picosecond timescale. Journal of Applied Physics 127, 204501 (2020).
[19] Zaffora, A. et al. Electrochemical Tantalum Oxide for Resistive Switching Memories. Advanced Materials 29, 1703357 (2017).
[20] Park, J. & Kim, S. Improving endurance and reliability by optimizing the alternating voltage in Pt/ZnO/TiN RRAM. Results in Physics 39, 105731 (2022).
[21] Yang, J. J. et al. High switching endurance in TaOx memristive devices. Applied Physics Letters 97, 232102 (2010).
[22] CRC Handbook of Chemistry and Physics. (CRC Press, 2016). doi:10.1201/9781315380476.

[23] Lecun, Y., Bottou, L., Bengio, Y. & Haffner, P. Gradient-based learning applied to document recognition. *Proc. IEEE* 86, 2278–2324 (1998).
[24] Xiao, H., Rasul, K. & Vollgraf, R. Fashion-MNIST: a Novel Image Dataset for Benchmarking Machine Learning Algorithms. Preprint at https://doi.org/10.48550/arXiv.1708.07747 (2017).
[25] Clanuwat, T. *et al.* Deep Learning for Classical Japanese Literature. Preprint at https://doi.org/10.48550/arXiv.1812.01718 (2018).
